# The Novel Role of hnRNP UL1 in Human Cell Nucleoli

**DOI:** 10.7150/ijbs.75084

**Published:** 2022-07-18

**Authors:** Marlena Cichocka, Anna Karlik, Patrycja Plewka, Kishor Gawade, Agata Stępień, Patrycja Świergiel, Ankur Gadgil, Katarzyna Dorota Raczyńska

**Affiliations:** 1 Laboratory of RNA Processing, Department of Gene Expression, Institute of Molecular Biology and Biotechnology, Faculty of Biology, Uniwersytetu Poznańskiego 6, 61-614 Poznan, Poland; 2 Center for Advanced Technology, Adam Mickiewicz University, Uniwersytetu Poznańskiego 10, 61-614 Poznan, Poland

**Keywords:** hnRNP UL1, nucleolus, rDNA genes, transcription, rDNA damage, rDNA repair

## Abstract

hnRNP UL1 plays an important role in cell nuclei, where it is recruited to DNA damage sites and is involved in the repair of DNA double-strand breaks. Furthermore, this protein is known as a transcriptional repressor of RNA polymerase II genes. In the present study, we have shown that hnRNP UL1 is also localized in the nucleoli of human cells. Upon investigating its function, we found that hnRNP UL1 stimulates ribosomal DNA (rDNA) gene transcription. Moreover, we observed that cells with hnRNP UL1 silencing exhibited increased sensitivity to DNA damage. We also showed that hnRNP UL1 interacts with γH2A.X, RPA32, XRCC1, and Chk1 in cell nucleoli, suggesting its involvement in the repair of rDNA damage.

## Introduction

The nucleolus is primarily the site of ribosomal RNA (rRNA) synthesis and ribosome assembly. It is the most transcriptionally active cellular organelle due to its repetitive sequences of ribosomal DNA (rDNA) genes 8. The high content of nucleic acids and ribosomal proteins creates a unique physical environment and influences the overall architecture of the nucleus. Sites containing rDNA genes are called nucleolar organizer regions (NORs) and are located on the short arms of acrocentric chromosomes 13, 14, 15, 21, and 22. The long intergenic spacer (IGS), which is 30 kb, contains regulatory elements that are located between rDNA units. Ribosomal RNAs are synthesized by RNA polymerase I (RNA Pol I). The rRNA gene is repetitively transcribed and generates a polycistronic unit called 47S pre-rRNA, which is further processed and modified into 18S, 5.8S, and 28S rRNA. In the next step of ribosome biogenesis, rRNAs are simultaneously assembled into large and small ribosomal subunits along with 5S rRNA 7,10,11,17,39,45,53,58,67. Both mutations and deletions of genes related to ribosome biogenesis, genes related to rRNA synthesis or modification, or genes encoding ribosome-related proteins can cause diseases termed "ribosomopathies" 16.

The organization of the nucleolus is linked to ribosome biogenesis and it can be divided into three subcompartments: i) the fibrillar center (FC), which contains factors associated with rRNA transcription, namely, RNA Pol I and factors such as DNA topoisomerase I and upstream binding factor (UBF); ii) the dense fibrillar component (DFC; the main site of pre-rRNA synthesis), which surrounds the FC and contains transcription factors, newly synthesized pre-rRNAs, and pre-rRNA processing factors; and iii) the granular component (GC), which surrounds the FC and DFC and contains ribosomal proteins, assembly factors, and nearly completed preribosomal subunits. The GC is the site of late processing events, when pre-rRNAs are assembled with ribosomal proteins to form ribosomal subunits. In addition, perinuclear heterochromatin (PH) surrounds the nucleolus. Heterochromatin plays an important role in nucleolar function by preventing homologous recombination between rDNA repeats, which in turn maintains nucleosome structure and rDNA stability 10,11,15,27,39,60.

Apart from its traditional role in ribosome biogenesis, the nucleolus is also involved in other cellular processes, including cell death, the cell cycle, proliferation, telomere metabolism, stress responses, energy production, DNA replication, recombination, and repair 11,21,32. Importantly, the nucleolar protein profile is dynamic; it can be modulated by changes in cell physiology during the cell cycle and may be influenced by stress, tumor development, signaling events and viral infections 1,14,46,48,52. In addition to rRNAs, the nucleolus also contains other types of RNAs, such as small nucleolar RNAs (snoRNAs). SnoRNAs are parts of small nucleolar ribonucleoprotein (snoRNP) complexes and catalyze rRNA posttranscriptional modifications and maturation. Spliceosomal U snRNAs and signal recognition particle (SRP) RNAs are also detected in the nucleolus. Other known RNAs, such as SRP RNA and RNase P RNA, also pass through the nucleolus 24.

Since ribosome production is a highly energy-intensive process, the function of the nucleolus is closely linked to cell growth and proliferation. In fact, almost all signaling pathways that affect these processes directly regulate rRNA synthesis. Additionally, the nucleolus monitors cellular stress signals and relays them to the RNA Pol I transcription machinery. As a result, rRNA synthesis is turned off to conserve energy, which is necessary to maintain cellular homeostasis. In response to a series of stresses, the nucleus activates a process called the nucleolar surveillance pathway (NSP), which results in the accumulation of the tumor suppressor protein p53. Elevated levels of p53 lead to impaired biogenesis and ribosome function, cell cycle arrest, and, in extreme cases, apoptotic cell death 43,64. Notably, nucleoli are also involved in cell differentiation and cancer transformation. Rapidly dividing cancer cells require an elevated rate of biogenesis and ribosome growth; hence, the size and number of nucleoli per cell are increased significantly 64.

The high level of rRNA transcription and the repetitive sequences of rDNA make rDNA susceptible to damage 61,66. rDNA is one of the most frequently rearranged chromosomal regions in tumorigenesis. The mechanisms of the nucleolar DNA damage response (n-DDR) in maintaining genome stability have become an antitumor research target 63. DNA double-strand breaks (DSBs) can arise as a consequence of replication stress or be induced by chemicals/enzymes or ionizing radiation (IR) 65. Cells have evolved two main pathways to respond to DSBs: nonhomologous end joining (NHEJ), a faster but mutagenic pathway involving DNA rejoining 54, and homologous recombination (HR), a slower pathway that requires homologous DNA sequences 28,62. The n-DDR and the consequences of transcription arrest by inhibition of RNA Pol I lead to nucleolar reorganization; that is, rDNA repeats and associated proteins move from the nucleolar interior to the periphery to form focal structures called "nucleolar caps" 17,39,40,56.

Heterogeneous nuclear ribonucleoprotein U-like protein 1 (hnRNP UL1), also known as adenovirus early region 1B-associated protein 5 (E1B-AP5), has been shown to play a role in transcription mainly by acting as a transcriptional regulator that inhibits gene expression; it also plays a role in splicing by directly binding to DNA and/or RNA or by interacting with other proteins in the spliceosome 4,18. It has also been reported that during cellular arrest, hnRNP UL1 represses the expression of replication-dependent histone genes in a complex with U7 snRNP 29. Moreover, hnRNP UL1 is involved in the cellular response to DNA damage. hnRNP UL1 directly interacts with p53 and inhibits its transcriptional activity in response to UV radiation 2. Moreover, hnRNP UL1 interacts with the MRN complex (MRE11, RAD50 and NBS1) *via* the NBS1 (Nibrin) protein. hnRNP UL1 together with the MRN complex and CtIP (C-terminal binding protein) is recruited to the site of DNA damage to participate in DSB repair 49. To be recruited to these sites, hnRNP UL1 must be methylated by protein arginine N-methyltransferase 1 (PRMT1) 22. hnRNP UL1 has also been reported to mediate the DSB damage response and/or repair in complex with poly [ADP-ribose] polymerase 1 (PARP1), one of the most important proteins required to maintain genome stability 26. Moreover, hnRNP UL1 is required for ataxia telangiectasia and Rad3-related protein (ATR)-dependent signaling in response to viral infection 2,26. In addition, hnRNP UL1 interacts with the long noncoding RNA (lncRNA), DNA damage-sensitive RNA1 (DDSR1), which affects cell proliferation, DDR signaling, and DNA repair capacity through HR. The interaction between DDSR1 and hnRNP UL1 regulates the ability of breast cancer type 1 susceptibility protein (BRCA1) and the BRCA1-A complex subunit RAP80 to access DSBs and thus modulates HR 55.

In this study, we showed that hnRNP UL1 can also be localized in the nucleoli of human cells. Upon investigating its potential function, we found that hnRNP UL1 stimulates the transcription of rDNA genes. Moreover, we noticed that cells with hnRNP UL1 knockout exhibited increased sensitivity to DNA damage, and the results suggest a role of hnRNP UL1 in rDNA repair pathways and nucleolar genome integrity. We confirmed that hnRNP UL1 interacts in cell nucleoli with phosphorylated histone H2AX (γH2A.X), replication protein A 32 kDa subunit (RPA32), X-ray repair cross-complementing protein 1 (XRCC1), and cell cycle checkpoint kinase (Chk1), suggesting its involvement in the repair of both DSBs and single-strand DNA (ssDNA) breaks.

## Materials and methods

### Cells

HeLa, HEK293T and HEK293 cells were grown in Dulbecco's modified Eagle medium (DMEM) with L-glutamine and 4.5 g/L glucose (Biowest) supplemented with 10% fetal bovine serum (Biowest) and antibiotics (100 U/ml penicillin, 100 µg/ml streptomycin, 0.25 µg/ml amphotericin B [Sigma]) at 37°C in a moist atmosphere containing 5% CO_2_. The cells were checked for mycoplasma, and only healthy cells were used for experiments. For DNA damage induction, the genotoxic reagents etoposide (ETO, type II topoisomerase inhibitor, Sigma #E1383) and camptothecin (CPT, type I topoisomerase inhibitor, Millipore #208925) diluted in DMSO were used. In the immunofluorescence experiment, cells were incubated with 10 µM ETO, 5 µM CPT or DMSO (as a control) for 2.5 h. For the Trypan blue assay 35, cells were treated with 0.5 μM ETO or 0.1 μM CPT. After 18, 24, 30, 48, 72, and 80 h, cells were detached with trypsin (General Chemistry Laboratory IITD PAN) and diluted 1:1 in Trypan blue (Invitrogen). Living cells were counted using a cell counter (Countess II, Invitrogen); the data are expressed as the percentage of cells in relation to the control. For the comet assay, cells were treated with 20 µM ETO and 10 µM CPT for 2.5 h before harvesting.

### Generation of a human cell line with *HNRNPUL1* knockout and overexpression

A CRISPR-Cas9 system based on efficient genome editing by the Cas9 nuclease was used to create a HEK293T cell line with knockout of the *HNRNPUL1* gene (HEK UL1 KO), according to the protocol already described by 51. For this purpose, two targets were selected using the CRISPR Design Tool (https://zlab.bio/guide-design-resources), and then oligonucleotides were designed for sgRNAs. The following oligonucleotides were used: hnRNPUL1-exon2Forward: 5'-ACCGAAAACGAGTCAGGCTACGAG-3'; hnRNPUL1-exon2Reverse: 5'-AAACCTCGTAGCCTGACTCGTTTTC-3'; hnRNPUL1-exon3Forward: 5'-CACCGTATGAAGAAAACCGGGGACG-3'; and hnRNPUL1-exon3Reverse: 5'-AAACCGTCCCCGGTTTTCTTCATAC-3'. The sequence overhangs used for ligation of the *BbsI* site pairs in PX458 (SpCas9-2A-EGFP, Addgene #48138) and PX459 (SpCas9-2A-Puro, Addgene #62988) are underlined. The primers used for PCR of the *HNRNPUL1* gene flanking the deletion region were as follows: forward: 5'-TCCGAGCTGGAGGGGACCGC-3'; reverse 5'-CCCTCCTATCCTCTCGGTGC-3'.

For transient expression, we used the pcDNA3.1 plasmid with a subcloned FLAG-hnRNP UL1 coding sequence, which was a kind gift from Prof. Stéphane Richard of the Terry Fox Molecular Oncology Group and Segal Cancer Center, McGill University, Montréal, Québec, Canada. The cells were transfected with Lipofectamine 2000 according to the manufacturer's instructions. To create a HEK293 cell line with stable expression of FLAG-hnRNP UL1 protein, a ready-to-use MultiMam™ Stable reagent kit (GENEVA BIOTECH) was used. The coding sequence of the *HNRNPUL1* gene extended with the FLAG sequence at the 5' end (in italics) was amplified using the following primers with restriction enzyme site overhangs (underlined): UL1.NheI.F: 5'-GCATCGCTAGCATG*GACTACAAAGACGATGACGACAAG*GATGTGCGCCGTC-3' and UL1.NsiI.R: - 5'-ACAGGATGCATCTACTACTACTGTGTACTTGTGCCA-3'. The product was ligated into the pMDS donor vector. Fusion to the pACEMam3/Integrator Module plasmid was performed by Cre-LoxP recombination. The pACEMam3 plasmid contains an FRT site, making it compatible with the Flp-In™-293 Cell Line (Life Technologies). HEK293 cells expressing FLAG-hnRNP UL1 (HEK UL1 OE cells) were prepared according to the manufacturer's protocol. The overexpression of hnRNP UL1 was confirmed by western blotting followed by immunostaining using anti-FLAG antibodies (Supplementary Fig. S1).

### Nucleolus isolation

Nucleolus isolation and nucleolar protein extraction were carried out according to the protocol described by 36. After fractionation, the following fractions were obtained: the nucleolar (NO) and cytoplasmic-nuclear (CN) fractions (Supplementary Fig. S2).

### Antibodies

In this work, the following antibodies were used: primary antibodies: anti-hnRNP UL1 (Abcam #ab68480 or Santa Cruz Biotechnology #sc-393434), anti-FLAG (Sigma #A8592), anti-Actin (MP #691001), anti-Fibrillarin (Santa Cruz Biotechnology #sc-25397), anti-Nucleolin (Abcam #ab22758), anti-FUS (Santa Cruz Biotechnology #sc-47711), anti-γH2A.X (Santa Cruz Biotechnology #sc-517348), anti-RPS6 (Abcam #ab70227), anti-RPS15 (Antikoerper #ABIN2786563), anti-RPA32 (Bethyl Laboratories #A300-245A), anti-pChk1 (Cell Signaling Technology #2341), anti-XRCC1 (Invitrogen #MA5-13412), anti-53BP1 (Abcam #ab175933), anti-Rad50 (Abcam #ab124682), anti-RPA194 (Santa Cruz Biotechnology #sc-46699), normal mouse IgG (Santa Cruz Biotechnology #sc-2025), and anti-digoxygenin-AP Fab fragments (Roche #11093274910); secondary antibodies: goat anti-mouse IgG-horseradish peroxidase (HRP) (Santa Cruz Biotechnology #sc-516102), goat anti-rabbit IgG-HRP (Santa Cruz Biotechnology #sc-2004), anti-mouse Alexa Fluor 555 (Thermo Fisher Scientific #A21422), anti-mouse Alexa Fluor 488 (Thermo Fisher Scientific #A32723), anti-rabbit Alexa Fluor 555 (Thermo Fisher Scientific #A32732) or anti-rabbit Alexa Fluor 488 (Thermo Fisher Scientific #A32731).

### Protein extraction, gel separation, western blotting and immunostaining

Protein extraction from whole cells (WCs), CN and NO fractions was performed with RIPA buffer (50 mM Tris-HCl, pH 8.0; 150 mM NaCl; 0.1% SDS; 0.5% sodium deoxycholate; 1% Triton X-100; 1 mM PMSF; cOmplete protease inhibitor cocktail tablets, EDTA-free). The samples were incubated for 15 min on ice and then centrifuged for 30 min at 4°C and 16,000× g. The supernatant containing protein extract was transferred to a fresh tube and used for further analysis. For SDS-polyacrylamide gel electrophoresis (SDS-PAGE), samples were mixed with gel loading buffer, denatured for 5 min at 95°C and then loaded on the gel. Electrophoresis was carried out with a 25 mA current. After electrophoresis, the proteins were transferred from the gel to a polyvinylidene difluoride (PVDF) membrane (Millipore) using a semidry system. After transfer, the membrane was blocked in 5% BSA overnight at 4°C and then incubated for 2 h at room temperature, first with primary antibodies and then with HRP-conjugated secondary antibodies. The signals were detected using an enhanced chemiluminescence (ECL) system (Healthcare).

### Immunoprecipitation (IP) and chromatin IP (ChIP)

For IP, the protein extracts from WCs and from the CN and NO fractions of wild-type HEK293 (HEK WT) or HEK UL1 OE cells were used. A total of 250 µg of protein extract isolated from HEK WT cells was incubated with Dynabeads Protein G conjugated with anti-hnRNP UL1 antibodies for 1.5 h at 4°C and then subjected to 3x 10 min washes in PBS with 0.02% Tween 20 (PBS-T) and eluted at 95°C for 10 min in 1x SSB buffer (50 mM Tris-HCl pH 6.8, 10% glycerol, 2% SDS, 10 mM DTT, 0.1% bromophenol blue). Protein G Dynabeads without antibodies were used as a negative control. A total of 250 µg of protein extract isolated from HEK UL1 OE cells was incubated overnight at 4°C with magnetic beads conjugated with anti-FLAG antibodies, washed five times with PBS-T and once with lysis buffer (50 mM Tris pH 7.8, 150 mM NaCl, 0.1% NP40) and eluted in 1x SSB buffer at 95°C for 10 min. Protein extract from HEK WT cells was used as a negative control. After elution, immunoprecipitated proteins were separated by SDS-PAGE, transferred to PVDF membranes, and detected by immunofluorescence.

In another approach, hnRNP UL1-interacting proteins were immunoprecipitated from HEK UL1 OE cells using μMACS™ Epitope Tag Protein Isolation Kits (MACS Molecular) according to the manufacturer's protocol. After IP, the proteins were precipitated by trichloroacetic acid (TCA), as follows: 100 μl of immunoprecipitated protein was precipitated by adding 50% TCA to a final concentration of 10% and Tween 20 to a final concentration of 0.5%. The mixture was incubated on ice for 30 min and then centrifuged for 10 min at 4,000 rpm at 4°C. Next, the pellet was washed once with cold 10% TCA and twice with cold 90% acetone. After each wash, the pellet was centrifuged for 10 min at 14,000 rpm at 4°C. After the last centrifugation, the supernatant was removed, and the pellet was air-dried at room temperature. Mass spectrometry analysis was performed in the Mass Spectrometry Laboratory, Institute of Biochemistry and Biophysics, Polish Academy of Science, Warsaw, Poland.

The ChIP experiment was performed as described by 19 using an antibody against RNA Pol I (anti-RPA194). The precipitated DNA was analyzed by quantitative PCR (qPCR) using the following gene-specific primer pairs: RDNA.PROMOTER.F, 5'-GGTATATCTTTCGCTCCGAG-3', and RDNA.PROMOTER.R 5'-AGCGACAGGTCGCCAGAGGA-3'; IGS.F, 5'-TGGTGGGATTGGTCTCTCTC-3', and IGS.R 5'-CAGCCTGCGTACTGTGAAAA-3'; RNA5S.F, 5'-CATACCACCCTGACGCG-3', and RNA5S.R, 5'-CTACAGCACCCGGTATTCCC-3'; RNA5.8S.F, 5'-ACTCGGCTCGTGCGTC-3', and RNA5.8S.R, 5'-GCGACGCTCAGACAGG-3'; RNA18S.F, 5'-GATGGTAGTCGCCGTGCC-3', and RNA18S.R, 5'-GCCTGCTGCCTTCCTTGG-3'; RNA28S.F, 5'-AGAGGTAAACGGGTGGGGTC-3', and RNA28S.R, 5'-GGGGTCGGGAGGAACGG-3'; RNA45S.F, 5'-GAACGGTGGTGTGTCGTT-3', and RNA45S.R, 5'-GCGTCTCGTCTCGTCTCACT-3'; and RNA47S.F, 5'-GTGCGTGTCAGGCGTTCT-3', and RNA47S.R 5'-GGGAGAGGAGCAGACGAG-3'.

### RNA isolation, cDNA synthesis, and qPCR

RNA was isolated using a Direct-zol^TM^ RNA MiniPrep Kit (ZYMO RESEARCH #R2052). First-strand cDNA was synthesized in the presence of random hexamers and Superscript III Reverse Transcriptase (SSIIIRT, Thermo Fisher) according to the manufacturer's protocol. Then, the cDNA template was diluted 4 times and used for qPCR amplification with gene-specific oligonucleotide primer pairs and Power SYBR Green PCR Master Mix (Applied Biosystems™ #4309155). qPCR was performed using a QuantStudio^TM^ 6 or 7 thermocycler (Thermo Fisher) with the following program: 95°C for 10 min and 40 cycles of 95°C for 15 s and 60°C for 1 min. The results of RT-qPCR analyses for rDNA gene expression in WCs and the CN fraction were normalized to the results for GAPDH, and those for the NO fraction were normalized to the results for U3 snoRNA. The statistical significance of the RT-qPCR results was determined by Student's t test.

### RNA-seq data preparation and analysis

RNA quality was checked using an Agilent Bioanalyzer 2100, and samples with an RNA integrity number (RIN) > 8.0 were used for library preparation. Five hundred nanograms of RNA was used for the preparation of the libraries without rRNA depletion. A CORALL Total RNA-Seq Library Preparation Kit (Lexogen, Vienna, Austria) was used to prepare libraries following the manufacturer's instructions. An Agilent High Sensitivity DNA Kit (Agilent, Santa Clara, CA, USA) was used to assess library quality on an Agilent Bioanalyzer 2100, and the libraries were quantified using a Qubit^TM^ dsDNA HS Assay Kit (Invitrogen^TM^). RNA sequencing was performed for 75 bp single reads using an Illumina NextSeq 500 platform at the Lexogen NGS facility (Vienna, Austria). The raw FASTQ files were processed. Briefly, the quality of the raw FASTQ files was assessed using fastqc/0.11.4 (http://www.bioinformatics.bbsrc.ac.uk/projects/fastqc). Cutadapt 3.7 41 was used with the default parameters to remove adapter sequences and low-quality reads from the FASTQ files. The processed FASTQ files from the previous step were aligned to the GRCh38 human genome assembly using the STAR/2.7.8a 13 aligner with the following parameters: outSAMattributes--All, outFilterType--BySJout, outFilterMultimapNmax--20, outFilterMismatchNmax--999, outFilterMismatchNoverLmax--0.04, and outFilterIntronMotifs--RemoveNoncanonical. A raw count matrix was obtained from the aligned BAM files in FeatureCounts 37. Differentially expressed genes (DEGs) were obtained with DESeq2 38 in the R package with the default parameters. DEGs with a P-adjusted (Padj) value less than 0.05 were considered significant. Volcano plots were generated using the R package ggplot2 68.

### Northern blot analysis

Northern blot analysis was performed as previously described 59 with some modifications. Briefly, 6 µg of total RNA was separated on a 1.2% denaturing agarose gel containing 1× H-E buffer (20 mM HEPES, 1 mM EDTA, pH 7.8) and 6% formaldehyde. Electrophoresis was performed in 1x H-E buffer at 55 V with recirculation for 7 h. After electrophoresis, the gel was subjected to mild alkaline treatment (10 min in 50 mM NaOH/10 mM NaCl), neutralization (10 min in 2.5x TBE) and equilibration in 2× SSC (0.3 M NaCl, 30 mM sodium citrate). The RNA was transferred overnight by capillary transfer to a Hybond N+ nylon membrane using 20× SSC (3 M NaCl, 0.3 M sodium citrate) and then immobilized on the membrane by UV crosslinking (1200x100 µJ/cm^2^). For detection of pre-rRNA processing intermediates, the membrane was first prehybridized in 10 ml of hybridization buffer (3.5% SDS, 0.375 M Na_2_HPO_4_ and 0.125 M Na_2_HPO_4_, 1% Blocking Reagent solution [Roche, 11096176001], 0.1 mg/ml poly[A]) for 1 h at 60°C. The prehybridization solution was removed, and 5 ml of fresh hybridization buffer was added along with a DIG-labeled DNA probe at a final concentration of 2 nM. The membrane was incubated with the probe for 1 h at 60°C and then overnight at 37°C. The HPLC-purified DNA probes were labeled using a DIG Oligonucleotide Tailing Kit (Roche, 03353583910) according to the manufacturer's protocol. The following day, the membrane was washed twice with 2x SSC/0.1% SDS and then six times with 0.2x SSC/0.1% SDS at the hybridization temperature. After washing, the membrane was subjected to immunodetection using anti-digoxigenin antibody, and the immunoreactive bands were visualized using the chemiluminescent substrate CDP-Star (Sigma-Aldrich, C0712) according to the manufacturer's protocol, with some modifications. Briefly, the membrane was first rinsed twice in 10 ml of washing buffer, blocked for 40 min in 10 ml of blocking solution, incubated with 10 ml of antibody solution (1:2500) for 1 h, washed four times in washing buffer, equilibrated for 5 min in detection buffer, incubated with CDP-Star substrate and then exposed to a luminescence imager. The following probes were used: ETS: 5'-CGGAGGCCCAACCTCTCCGACGACGACAGGTCGCCAGAGGACAGCGTG-3', ITS1: 5'-CCTCGCCCTCCGGGCTCCGTTAATGATC-3', and ITS2: 5'-CTGCGAGGGAACCCCCAGCCGCGCA-3' 33.

### Microscopic analysis

For immunostaining, cells were cultured on 8-well µSlides (50 000 cells/well), fixed with 4% paraformaldehyde (PFA) for 12 min, washed in PBS, permeabilized in 1x PBS + 0.5% Triton X-100 for 15 min and washed again in PBS. The cells were then incubated in blocking solution (1% BSA in PBS) for 30 min and then incubated with primary antibodies diluted 1:200 in blocking solution for 1 h. The cells were then washed 3x for 10 min in PBS and incubated with secondary antibodies diluted 1:200 in blocking solution for 45 min. Finally, the cells were washed 3x for 10 min in PBS to remove any unbound antibodies, stained with DAPI (Thermo Fisher Scientific) for 10 min and postfixed with 4% PFA for 5 min. Images were acquired using an Olympus Fluoview 1200 IX83 confocal scanning microscope with a 60x oil-immersion objective. Three channels were used to acquire images with the following excitation parameters: 488 nm for Alexa Fluor 488, 559 nm for Alexa Fluor 555 and 405 nm for DAPI. The obtained images were analyzed using ImageJ software.

### Comet assay

For DNA damage measurement, a comet assay kit (Abcam #ab238544) was used according to the manufacturer's protocol. Images were acquired using an Olympus Fluoview 1200 IX83 confocal scanning microscope with a FITC filter. CASPlab software was used to analyze the results. Two parameters were analyzed, the tail DNA % and tail moment. The tail DNA % was calculated as follows: tail DNA % = 100x tail DNA intensity/cell DNA intensity. The tail moment was measured as the Olive Tail Moment (OTM): OTM = tail DNA% x tail moment length. Fifty cells were analyzed for each sample. The statistical significance was determined by Student's t test.

### Polysome profiling

The experiment was performed in three biological replicates, according to the protocol adapted from 9.

## Results

### hnRNP UL1 interacts with ribosomal proteins and affects rRNA levels

**Figure 1:**
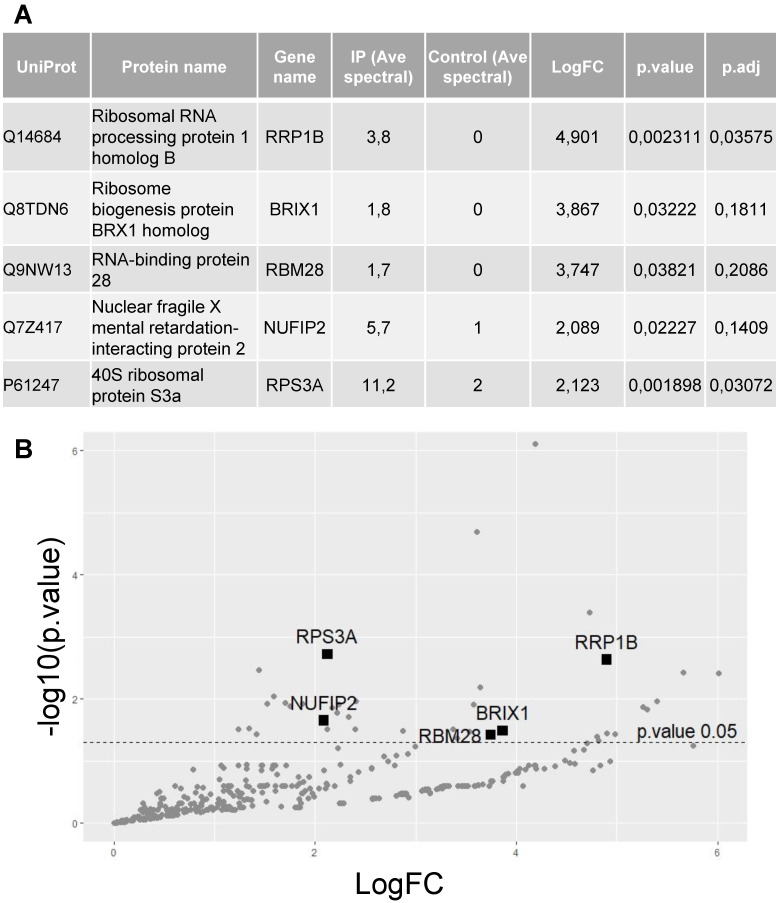
** Identification of proteins that interact with hnRNP UL1**. (A) Protein extract from HEK293T cells overexpressing FLAG-hnRNP UL1 was used for immunoprecipitation followed by mass spectrometry analysis. IP (Ave spectral) and control (Ave spectral) are the average numbers of spectra detected for a given protein for IP and control, respectively. (B) Volcano plot showing proteins from WC extracts; the 5 proteins described in (A) are highlighted.

To identify proteins interacting with hnRNP UL1, a plasmid encoding FLAG-hnRNP UL1 was transiently expressed in HEK293T cells (Supplementary Fig. S1). Twenty-four hours after transfection, whole-cell protein extract was isolated and used for immunoprecipitation with an anti-FLAG antibody; nontransfected cells were used as controls. Immunoprecipitated proteins were identified by mass spectrometry. Interestingly, among hnRNP UL1-interacting factors, we found 5 proteins that were defined as ribosomal proteins and/or proteins involved in ribosomal assembly (Fig. 1A, B). Furthermore, all selected proteins exhibit nucleolar localization. They include rRNA processing protein 1 homolog B (RRP1B), ribosome biogenesis protein BRX1 homolog (BXDC2/BRX1), RNA-binding protein 28 (RBM28), nuclear fragile X mental retardation-interacting protein 2 (NUFIP2) and 40S ribosomal protein S3a (RPS3A).

**Figure 2:**
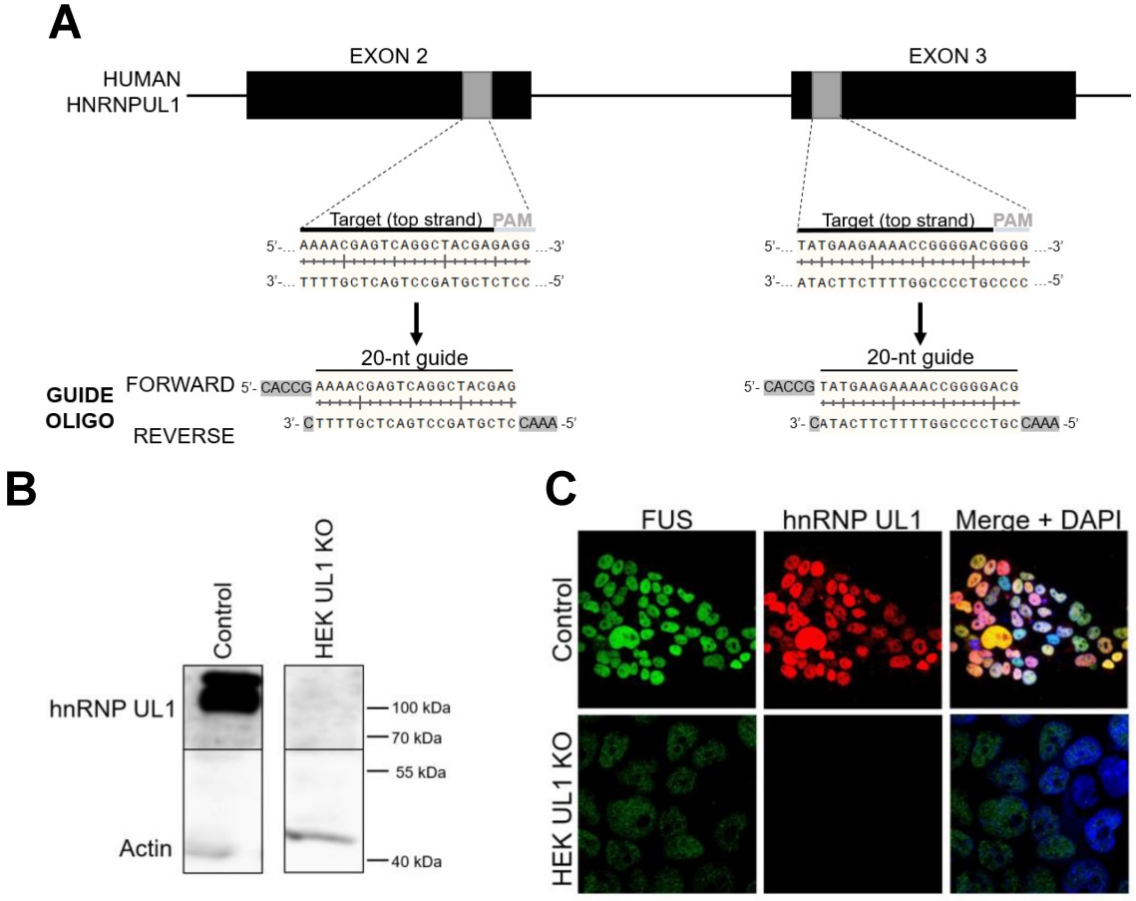
** Generation of cells with hnRNP UL1 knockout.** (A) The CRISPR-Cas9 system was used to prepare HEK UL1 KO cells. Two targets located on exons 2 and 3 were selected (gray boxes). The 20-bp target sequences (black line) are preceded at the 3' end by 5'-NGG protospacer-adjacent motifs (PAMs) (gray line), where N stands for any nucleotide. The two designed oligonucleotide guides (forward and reverse) contain overhangs (highlighted in gray) needed for ligation into pSpCas9-2A-EGFP or pSpCas9-2A-Puro plasmids using *BbsI* restriction sites. (B) Western blot analysis followed by immunostaining and (C) immunofluorescence using an anti-hnRNP UL1 antibody was performed to confirm the silencing of hnRNP UL1 protein in HEK UL1 KO cells in comparison to HEK WT cells (control). Actin and FUS were used as controls. DAPI was used for nuclear staining.

To identify transcripts that can be affected by hnRNP UL1, we performed high-throughput sequencing of RNA (RNA-seq) isolated from HEK293T cell line with knockout of the *HNRNPUL1* gene (HEK UL1 KO cell line), which was created using the CRISPR-Cas9 system based on efficient genome editing by Cas9 nuclease (Fig. 2A). To verify the effectiveness of the strategy, a part of the sequence encompassing the deletion region was analyzed using primers designed several base pairs upstream and downstream of the cut site (Supplementary Fig. S3). Silencing of *HNRNPUL1* gene expression was further confirmed at the protein level by western blotting followed by immunostaining and immunofluorescence using anti-hnRNP UL1 antibodies (Fig. 2B and C). For RNA-seq, because previous results have shown that hnRNP UL1 interacts with nucleolus-localized proteins, we decided to use RNA isolated from cells fractionated into CN and NO fractions without an rRNA depletion step (Supplementary Fig. S2). The results from HEK UL1 KO cells were compared to those from HEK WT cells. As shown in Table 1 and Supplementary Table S1, in both the CN and NO fractions, we observed significantly lower 5S, 5.8S, 18S, 28S and 45S rRNA levels in HEK UL1 KO cells than in HEK WT cells. In summary, both the RNA-seq and IP results suggest that hnRNP UL1 might be involved in rDNA gene transcription and/or ribosome biogenesis.

### hnRNP UL1 is localized in human nucleoli and participates in rDNA gene transcription

**Figure 3:**
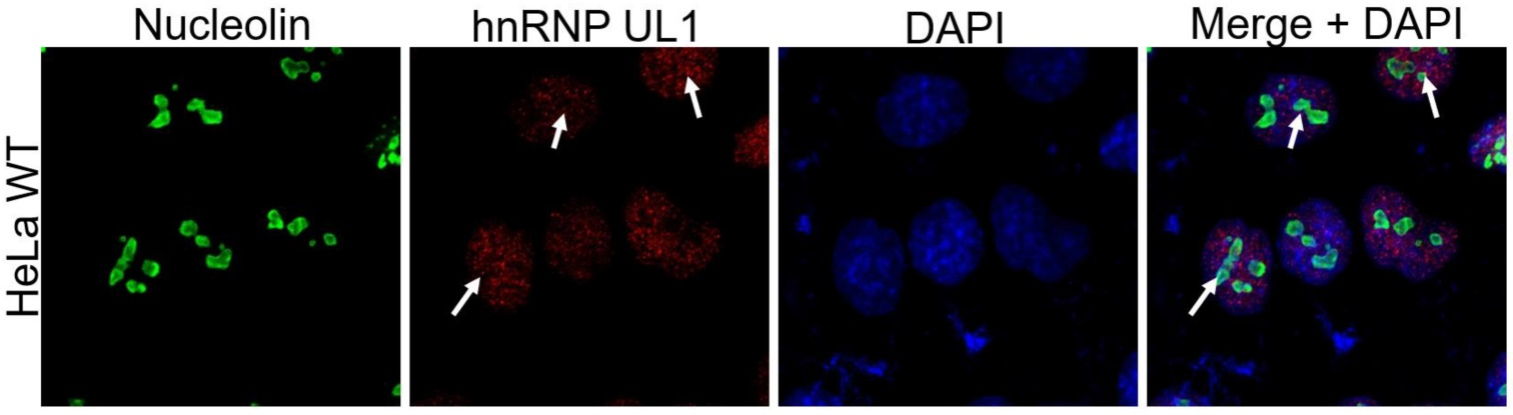
** Localization of hnRNP UL1 in the nucleoli of human cells**. Immunostaining was performed in HeLa WT cells using antibodies against nucleolin and hnRNP UL1. Sites of colocalization are indicated by white arrows. DAPI was used for nuclear staining.

As rDNA gene transcription and ribosome biogenesis take place in the nucleolus, we tested whether hnRNP UL1 can localize in this nuclear region. As shown in Fig. 3, immunofluorescence experiments confirmed that hnRNP UL1 colocalizes with nucleolin in the nucleoli of human cells.

**Figure 4:**
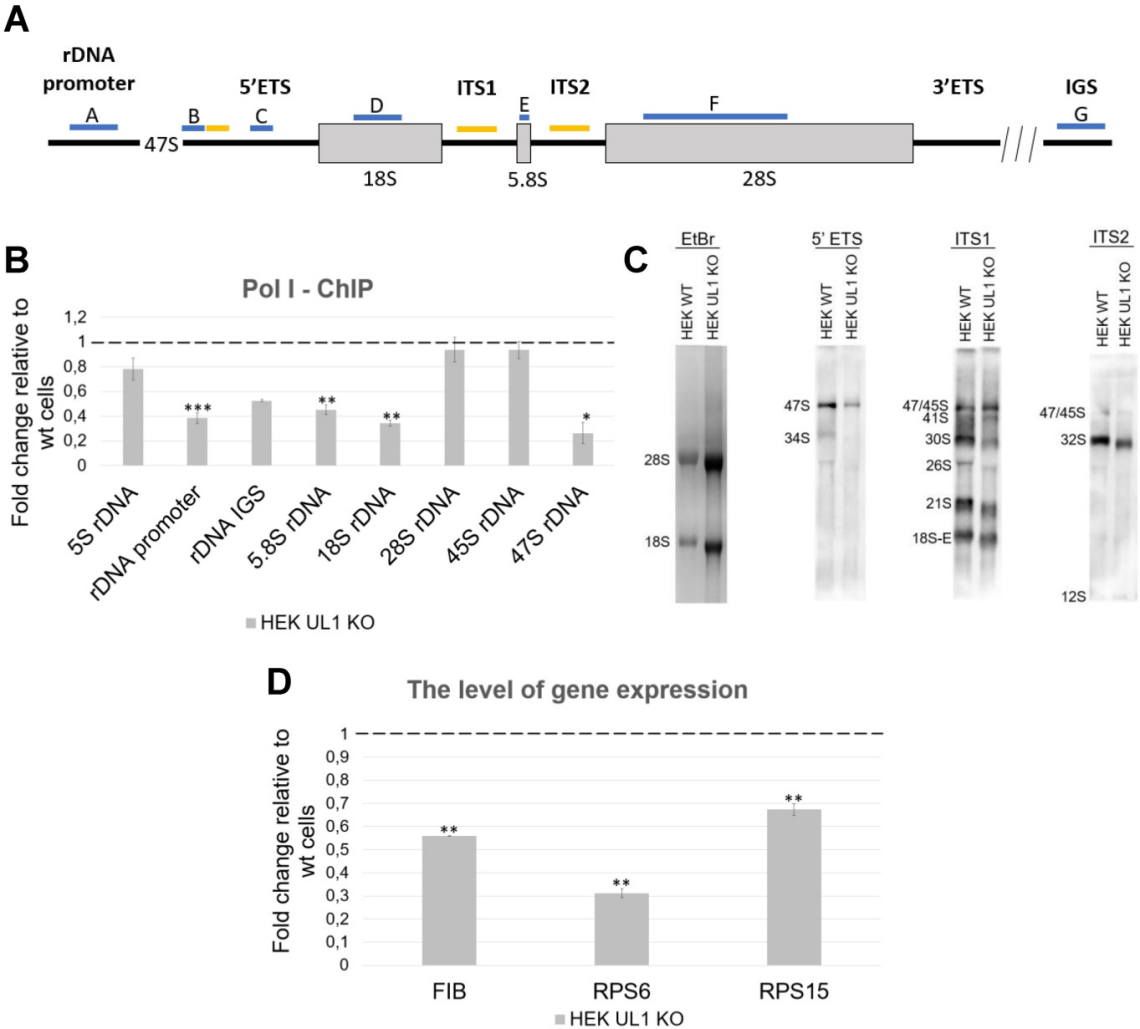
** Regulation of the expression of rDNA genes and ribosomal protein genes by hnRNP UL1**. (A) Scheme showing the 47S pre-rRNA with the positions of the primers and probes used in qPCR and northern blotting, respectively. The sites of PCR primer pairs are indicated in blue: A - on rDNA promoter, B - on 47S rDNA, C - on 45S rDNA, D - on 18S rDNA, E - on 5.8S rDNA, F - on 28S rDNA, and G - on IGS rDNA. Yellow indicates probes for northern blotting: 5'ETS, ITS1 and ITS2. (B) RNA Pol I binding at different regions of the rDNA loci (promoter, IGS, 5.8S, 18S, 28S, 45S, and 47S) was quantified by ChIP combined with qPCR in HEK UL1 KO cells compared to HEK WT cells. 5S rRNA was used as a negative control. (C) Total RNA extracted from HEK WT and HEK UL1 KO cells was separated on a denaturing gel and analyzed by northern blotting. The blots were probed with the oligonucleotides 5'ETS, ITS1 and ITS2. The detected pre-rRNA species are highlighted. (D) The mRNA levels of two ribosomal proteins (RPS6 and RPS15) and the nucleolar marker fibrillarin (FIB) were analyzed by RT-qPCR in HEK UL1 KO cells compared to HEK WT cells. The error bars represent the SDs of three biological replicates. P values were calculated using Student's t test, and the statistical significance is represented as follows: *P ≤ 0.05; **P ≤ 0.01; ***P ≤ 0.001.

Given this observation and previous results from RNA-seq, we concluded that hnRNP UL1 might be involved in pre-rRNA synthesis and/or maturation in the nucleolus. Therefore, in the next step, we tested the transcription efficiency of rDNA genes and the levels of mature rRNAs (5S, 5.8S, 18S, and 28S) and their precursors (45S and 47S) by ChIP, Northern blotting, and RT-qPCR. The ChIP assay was performed on WC isolated from HEK WT and HEK UL1 KO cells using anti-RNA Pol I antibody followed by qPCR with primers designed to amplify different regions of the rDNA gene locus (Fig. 4A). As shown in Fig. 4B, knockout of hnRNP UL1 diminished the binding of RNA Pol I at both the rDNA promoter region and the 5.8S, 18S, and 47S rDNA regions. These results indicate that hnRNP UL1 may play a role in rDNA transcription and mediate RNA Pol I binding to rDNA gene loci in human nucleoli, leading to transcriptional repression in cells with hnRNP UL1 knockout. Next, a pre-rRNA processing analysis was performed *via* Northern blotting with probes detecting major known pre-rRNA processing intermediates. Consistent with the ChIP-qPCR results, we observed reduced accumulation of 47S primary transcripts and consequent decreases in the levels of downstream processing intermediates (30S, 26S, 21S, 18S-E rRNAs) in HEK UL1 KO cells (Fig. 4C). For RT-qPCR analysis, cDNA was prepared from RNA isolated from WCs as well as from NO and CN fractions of HEK WT and HEK UL1 KO cells (Supplementary Fig. S4A-C). As shown in Supplementary Fig. S4C, the changes were mostly apparent in the nucleolar fraction, with the levels of 47S and 28S rRNAs significantly decreased in HEK UL1 KO cells. A decreased level of 5.8S rRNA was observed in the WC fraction (Supplementary Fig. S4A), whereas the level of 18S rRNA was not reproducible.

Furthermore, we also checked the expression of exemplary ribosomal protein genes encoding fibrillarin, RPS6 and RPS15 in HEK WT and HEK UL1 KO cells. As shown in Fig. 4D, the levels of all three mRNAs were downregulated. However, we did not observe any changes in their expression at the protein level (Supplementary Fig. S5). Next, we performed polysome profiling in HEK WT and HEK UL1 KO cells; however, we did not observe any changes in either ribosome or polysome profiles after hnRNP UL1 depletion (Supplementary Fig. S6). All these results indicate that hnRNP UL1 is involved in rDNA gene transcription but not in the subsequent steps of ribosome biogenesis.

### hnRNP UL1 plays a role in rDNA damage repair in nucleoli of human cells

**Figure 5:**
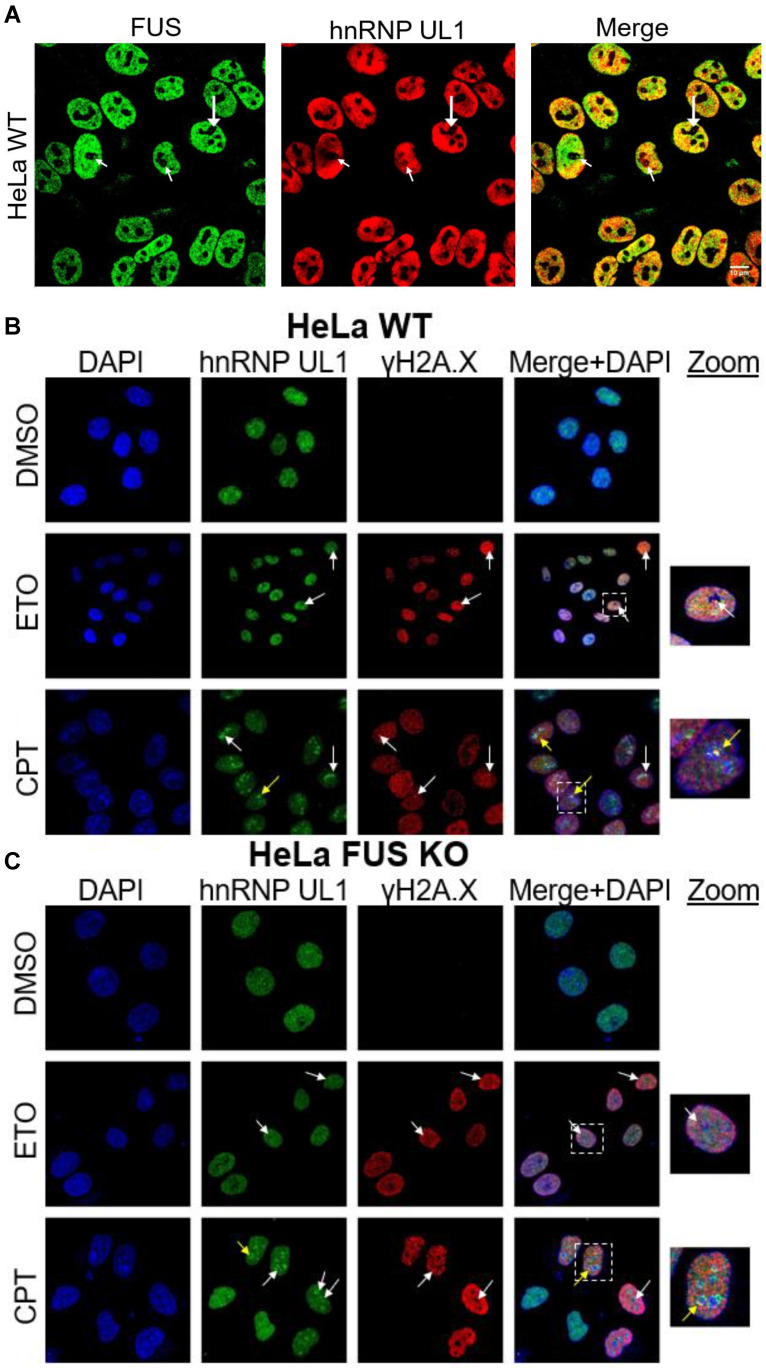
** Colocalization of hnRNP UL1 and γH2A.X after DNA damage induction**. (A) Colocalization of hnRNP UL1 and FUS in human nucleoli was tested by immunofluorescence using anti-FUS and anti-hnRNP UL1 antibodies. Exemplary sites of colocalization are indicated by white arrows. (B, C) Immunostaining using anti-γH2A.X and anti-hnRNP UL1 antibodies in HeLa WT (B) and HeLa FUS KO (C) cells treated with ETO and CPT. Cells treated with DMSO were used as controls. Exemplary sites of colocalization are indicated by white arrows, and aggregates in the periphery are indicated by yellow arrows. DAPI was used for nuclear staining. Scale bars: 20 μm.

As reported previously, hnRNP UL1 participates in DNA damage repair in the nuclei of human cells, by interacting, among others, with p53, the MRN complex (*via* NBS1), PARP1 and DDSR1 2,22,26,34,49,55. Our observation that hnRNP UL1 is localized in the nucleolus prompted us to hypothesize that the protein also engages in rDNA damage repair in human nucleoli. To test this hypothesis, we first assessed the levels of the DNA damage marker γH2A.X in HEK UL1 KO cells. However, we did not observe any significant changes, indicating that cells with hnRNP UL1 depletion do not generate more DNA damage than those with intact hnRNP UL1 (Supplementary Fig. S7). Next, we analyzed the colocalization of both proteins in mammalian cell nucleoli under control conditions (DMSO) and after treatment with the genotoxic reagents ETO (10 µM) and CPT (5 µM) for 2.5 h. Additionally, hnRNP UL1 and γH2A.X colocalization was tested under the same conditions in HeLa cells with depletion of FUS (HeLa FUS KO cells); FUS has been recently reported to be relocated to nucleoli in response to DNA damage 42. FUS and hnRNP UL1 are known to interact with each other 50 and to colocalize in the cell nucleoli (Fig. 5A). As shown in Fig. 5B, after ETO- and CPT-induced DNA damage, hnRNP UL1 and γH2A.X colocalized mainly in the nucleus; however, after treatment with CPT, both factors strongly aggregated in the nucleolus (as indicated by the white arrows), especially in the periphery (as indicated by the yellow arrow). Such localization of these proteins after DNA damage may indicate their participation in DSB repair and recruitment to the nucleolar caps. Interestingly, no obvious differences were observed in HeLa FUS KO cells compared to HeLa WT cells (Fig. 5C), indicating that FUS depletion does not affect the localization of hnRNP UL1 in the nucleolus.

**Figure 6:**
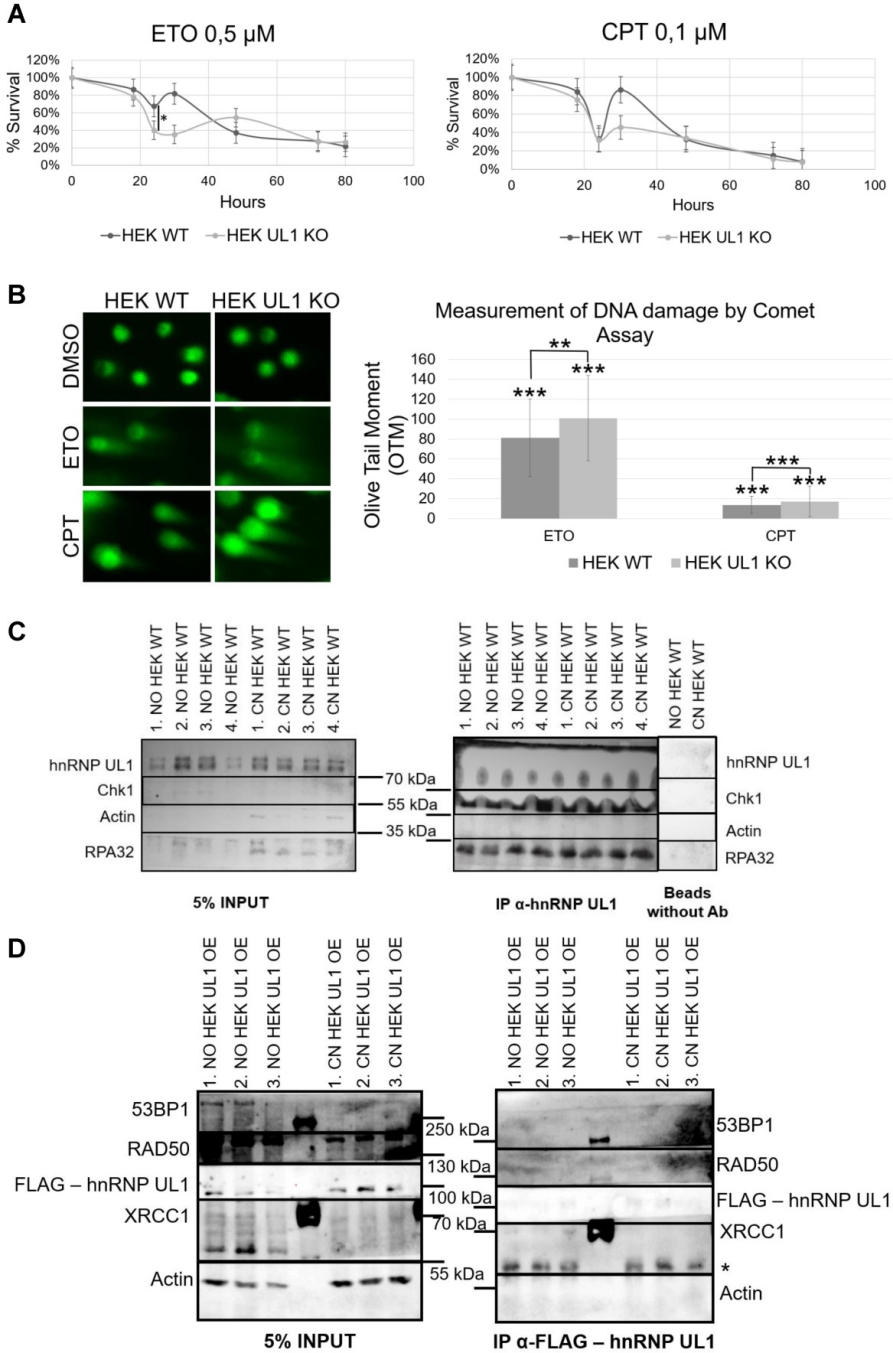
** Involvement of hnRNP UL1 in the DDR in human nucleoli.** (A) The DNA damage sensitivity of HEK UL1 KO cells compared to HEK WT cells was tested after 2.5 h of treatment with the genotoxic reagents ETO and CPT. After this time, the cells were harvested at the following time points: 18 h, 24 h, 30 h, 48 h, 72 h and 80 h. Cell viability was assessed by Trypan blue staining, and the percentage of survival was calculated. The results were normalized to those for the control cells treated with DMSO. (B) DNA damage was assessed by Comet Assay in HEK UL1 KO cells compared to HEK WT cells after DNA damage induced with ETO and CPT. Cells treated with DMSO were used as negative controls. For the assay, 50 cells per sample were analyzed. The error bars represent the SDs of three biological replicates. The p values were calculated using Student's t test, and the statistical significance is represented as follows: *P ≤ 0.05; **P ≤ 0.01; ***P ≤ 0.001. (C) IP was performed using an anti-hnRNP UL1 antibody and protein extracts from the NO and CN fractions of HEK WT cells. After elution, the immunoprecipitated proteins were identified by western blotting followed by immunostaining. Four independent biological replicates from each fraction were used for the experiment. For the input, 5% of the total volume applied to the beads was used; for the negative controls, beads without antibody were used; for immunostaining, anti-hnRNP UL1, anti-pChk1, and anti-pRPA32 antibodies were used; and actin was used as a loading control. (D) IP was performed using magnetic beads conjugated with an anti-FLAG antibody and protein extracts from the NO and CN fractions of HEK UL1 OE cells. Three independent biological replicates from each fraction were used for the experiment. For the input, 5% of the total volume applied to the beads was used. For immunostaining, anti-53BP1, anti-RAD50, anti-FLAG, and anti-XRCC1 were used, and an anti-actin antibody was used as a control. * - nonspecific signal.

Next, we tested whether depletion of UL1 results in DNA damage sensitization. For this purpose, the sensitivity of HEK UL1 KO cells to the DNA damage induced by the genotoxic reagents ETO and CPT was compared to the sensitivity of HEK WT cells. As shown in Fig. 6A, cells with hnRNP UL1 depletion were more sensitive to both reagents than WT cells, reaching the minimal survival point and exhibiting the greatest difference after 24-30 h. After that time, the sensitivity of both kinds of cells became comparable again. This result suggests that cells with knockout of hnRNP UL1 exhibit increased sensitivity to DNA damage and a potentially slowed repair process, confirming a role for hnRNP UL1 in DNA damage repair and genome integrity.

In another approach, we performed a comet assay to measure ETO- and CPT-induced DNA damage in HEK UL1 KO cells and HEK WT cells compared to cells treated with DMSO. Two parameters were used for calculations: the tail DNA% and tail moment, which are suitable indicators of induced DNA damage considering both the migration of genetic material and the relative amount of DNA in the tail. As shown in Fig. 6B, among cells treated with both ETO and CPT, the comet tails were longer in HEK UL1 KO cells than in HEK WT cells, which was also confirmed by calculations. This observation confirms that cells with hnRNP UL1 knockdown exhibit increased susceptibility to DNA damage.

Furthermore, to test whether hnRNP UL1 can directly participate in DNA repair in the nucleoli, we performed an IP experiment using an anti-hnRNP UL1 antibody and the NO and CN fractions of HEK WT cells, followed by western blot analysis and immunodetection with anti-hnRNP UL1, anti-pChk1, and anti-pRPA32 antibodies. RPA32 is a part of the RPA complex, which recognizes single-stranded DNA breaks and is one of the major complexes involved in the DNA repair pathway in both the nucleus and the nucleolus. Chk1 kinase plays an important role in DNA damage checkpoint control. As shown in Fig. 6C, both proteins interacted with hnRNP UL1 in both the NO and CN fractions of HEK WT cells. To estimate the exact pathways of DDR in which hnRNP UL1 is involved, we performed IP using anti-hnRNP UL1 and anti-FLAG antibodies in HEK WT and HEK UL1 OE cells, respectively. For this purpose, we created a HEK293 cell line with stable expression of FLAG-hnRNP UL1 using a ready-to-use MultiMam™ Stable system (Supplementary Fig. S1). We tested the interaction of hnRNP UL1 with 53BP1, RAD50, RPA32 and XRCC1. 53BP1 is involved in the signaling and repair of DNA DSBs in human cells; RAD50 belongs to the MRN complex; and XRCC1 participates in the repair of DNA single-strand breaks by mediating the formation of protein complexes that repair DNA damage. As shown in Fig. 6D, in this experiment, only a weak interaction of hnRNP UL1 with XRCC1 in the NO fraction was observed. However, immunofluorescence using anti-hnRNP UL1 and anti-XRCC1 antibodies performed in HEK WT cells after DNA damage induction showed no clear colocalization of this protein with hnRNP UL1 in the cell nucleoli (Supplementary Fig. S8A). Moreover, little colocalization of hnRNP UL1 and 53BP1 or RAD50 and RPA32 in the nucleolus was observed in this experiment (Supplementary Fig. S8B-D).

## Discussion

We report, for the first time, that hnRNP UL1 is localized in the nucleoli of human cells (Fig. 3). Knockout of the *HNRNPUL1* gene results in altered expression of rDNA genes, with 28S and 47S rRNA levels significantly decreased in the NO fraction, suggesting the role of hnRNP UL1 in rRNA synthesis and/or maturation in the nucleolus (Fig. 4A, Supplementary Fig. S4C). Furthermore, we found that hnRNP UL1 influences RNA Pol I recruitment to the rDNA promoters and 5.8S, 18S, and 47S rDNA regions, thereby indicating its function in RNA Pol I binding to rDNA genes and transcription in the nucleolus (Fig. 4B). In addition, we observed that hnRNP UL1 interacts with ribosomal proteins (Fig. 1) and may affect expression of ribosomal protein genes (Fig. 4D), although in other experiments, we found that hnRNP UL1 depletion did change neither nucleolar/ribosomal protein levels (Supplementary Fig. S5) nor polysome abundance (Fig. S6). Therefore, we suggest that hnRNP UL1 may be involved in the transport of ribosomal proteins or their posttranslational modifications rather than in ribosome biogenesis. However, to confirm this hypothesis, more studies need to be performed. Notably, the lack of mRNA-protein expression correspondence is not surprising given the essential roles of ribosomal proteins in cell growth. It has been previously reported that transcriptional regulation does not play a considerable role in ribosomal protein production, which appears to be mainly regulated at translational and posttranslational levels to adjust the biosynthesis of the ribosomes to the specific requirements of the cells 5. Therefore, diminished transcript levels can be equilibrated with increased translation or protein stability.

In agreement with previous research reporting the role of hnRNP UL1 in DNA damage repair in the nucleus, we found that hnRNP UL1 may also be involved in n-DDR. We observed that cells with hnRNP UL1 knockout exhibited increased sensitivity to DNA damage, resulting in an increased number of dead cells. Similar results have been obtained for cells with silencing of Treacle and MRE11, which are key proteins involved in n-DDR and the HR repair pathway 30,31,44. Moreover, we confirmed that hnRNP UL1 can interact in the nucleolus with proteins involved in different pathways of DNA break repair (Fig. 6C-D).

In this study, we examined the interaction in the nucleolus of hnRNP UL1 with selected proteins that are known to be localized in DNA damage sites and involved in DNA repair mechanisms. The first was γH2A.X. This phosphorylated histone protein is essential for cell cycle arrest and DNA damage repair after DSBs 20. In transcribed rDNA, histone H2A.X has reduced nucleosome occupancy, so upon ATM activation, limited phosphorylation of H2A.X is detected 23,31. The second was 53BP1, which plays a key role in the repair of DSBs by promoting NHEJ and specifically counteracting the function of the BRCA1 HR repair protein 6. The third, RAD50, is a member of the MRN complex, which is a major complex involved in the HR repair pathway of DSBs in both the nucleus and nucleolus 12. Fourth, RPA32, also known as RPA2, is a member of the RPA complex that recognizes ssDNA and is one of the major complexes involved in the DNA repair pathway in both the nucleus and nucleolus. The RPA complex, by recruiting ATRIP, activates ATR kinase, a master regulator of the DDR that is required for the recruitment of the DSB repair factors RAD51 and RAD52. It also recruits proteins such as endonucleases XPA and XPG, which are involved in DNA repair by nucleotide excision 57. Fifth was XRCC1, which participates in ssDNA damage repair by mediating the formation of DDR protein complexes. XRCC1 negatively regulates ADP-ribose levels by modulating ADP-ribosyltransferase PARP1 activity 25. Sixth was Chk1, a serine/threonine-protein kinase that is required for cell cycle arrest and activation of DNA repair after DSBs. Chk1 is activated as part of the n-DDR 44. According to our observations, hnRNP UL1 interacts in the nucleolar fraction with γH2A.X, RPA32, XRCC1, and Chk1 (Fig. 5B-C, Fig. 6C-D), suggesting that it may be localized in the sites of rDNA damage and participate in the repair pathways in the nucleolus. This is the first report that hnRNP UL1 might be involved in ssDNA break repair in the nucleolus *via* interaction with RPA32 and XRCC1.

Interestingly, after DNA damage induction with the reagent CPT, we observed in both HeLa WT and HeLa FUS KO cells a co-localization of hnRNP UL1 with γH2A.X in the nucleoli, particularly at the periphery of the nucleoli. This may suggest that hnRNP UL1 is recruited to nucleolar caps after DSBs and that it mediates the HR repair pathway. hnRNP UL1 can also cooperate with Chk1 in rDNA repair after DSBs. It has already been shown that hnRNP UL1 and hnRNP UL2 together stimulate DNA end resection and promote ATR-dependent signaling and DSB repair by HR, affecting cell viability. In the nucleus, hnRNP UL1 binds to NBS1, which is a part of the MRN complex, to play a role in the cellular response to DSBs in the nucleus. In this work, we tried to detect the interaction of hnRNP UL1 with another subunit of the MRN complex, RAD50, in human nucleoli. However, we did not observe specific interactions between hnRNP UL1 and RAD50 in either the NO or CN fractions. The hnRNP UL1 protein also functions downstream of MRN and CtIP to promote BLM helicase recruitment to DNA damage sites. Recruitment of hnRNP UL1 to DSBs is dependent on the MRN complex and PARP1 2,3,22,26,49,55.

In light of recent findings, the FUS protein is required for the recruitment of DDR factors to DNA damage sites; moreover, FUS-dependent liquid-liquid phase separation (LLPS) is essential for DDR activation and proper formation of DSB repair complexes in the nucleus 35. Furthermore, FUS has been shown to change localization after induction of DNA breaks by topoisomerase type I (TOP1), and it localizes in the nucleolus in response to RNA polymerase II inhibition 42. In our experiments, silencing of FUS had no significant effect on hnRNP UL1 localization in the nucleolus (Fig. 5C).

In summary, our research provides insights into the putative role of hnRNP UL1 in the nucleoli of human cells, suggesting two possibilities: hnRNP UL1 functions as an activator of rDNA gene transcription and ii) hnRNP UL1 is one of the factors involved in n-DDR for both single-strand DNA breaks and DSBs, including the HR DSB repair pathway. Such an observation broadens our knowledge about the response to rDNA damage in the nucleolus, where the availability of DNA repair factors is limited. However, the details of the existing mechanisms remain to be elucidated.

## Supplementary Material

Supplementary figures and table.

## Figures and Tables

**Table 1:** ** The expression of rDNA genes is affected in hnRNP UL1 knockout cells.** Selected transcripts represent 5S, 5.8S, 18S, 28S and 45S rRNAs that were significantly (p value<0.05) changed in the CN and NO fractions in HEK UL1 KO cells relative to HEK WT cells.

GeneID	baseMean	Fold Change	p.value	p.adj	Fraction
RNA5-8SP8	92224,52912	0,300	0,000	0,000	CN
RNA5SP74	111,7623806	0,378	0,005	0,020	CN
RNA5-8SN3	52744,81405	0,526	0,030	0,202	NO
RNA5SP506	310,3483873	0,546	0,020	0,165	NO
RNA18SN3_1	1329605,513	0,597	0,033	0,210	NO
RNA45SN3_1	2388683,306	0,615	0,036	0,219	NO
RNA28SN1	1019822,894	0,624	0,035	0,217	NO
RNA18SN3	2506339,157	0,738	0,005	0,020	CN
RNA45SN3_1	4109132,44	0,793	0,021	0,068	CN

## Abbreviations

hnRNP UL1: heterogeneous nuclear ribonucleoprotein U-like protein 1
rRNA: ribosomal RNA
rDNA: ribosomal DNA
NORs: nucleolar organizer regions
IGS: long intergenic spacer
RNA Pol I: RNA polymerase I
FC: fibrillar center
UBF: upstream binding factor
DFC: dense fibrillar component
GC: granular component
PH: perinuclear heterochromatin
snoRNAs: small nucleolar RNAs
snoRNP: small nucleolar ribonucleoprotein
SRP: signal recognition particle
NSP: nucleolar surveillance pathway
DSBs: DNA double-strand breaks
n-DDR: nucleolar DNA damage response
IR: ionizing radiation
NHEJ: nonhomologous end joining
HR: homologous recombination
E1B-AP5: adenovirus early region 1B-associated protein 5
NBS1: Nibrin
CtIP: C-terminal binding protein
PRMT1: arginine N-methyltransferase 1
PARP1: poly [ADP-ribose] polymerase 1
ATR: ataxia telangiectasia and Rad3-related protein
lncRNA: long noncoding RNA
DDSR1: DNA damage-sensitive RNA1
BRCA1: breast cancer type 1 susceptibility protein
γH2A.X: phosphorylated histone H2AX
RPA32: replication protein A 32 kDa subunit
XRCC1: X-ray repair cross-complementing protein 1
Chk1: cell cycle checkpoint kinase
DMEM: Dulbecco's modified Eagle medium
ETO: etoposide
CPT: camptothecin
HEK UL1 KO: HEK293T cell line with knockout of the *HNRNPUL1* gene
HEK UL1 OE: HEK293 cells expressing FLAG-hnRNP UL1
NO: nucleolar
CN: cytoplasmic-nuclear
WCs: whole cells
SDS-PAGE: SDS-polyacrylamide gel electrophoresis
PVDF: polyvinylidene difluoride
ECL: enhanced chemiluminescence
IP: immunoprecipitation
ChIP: chromatin IP
HEK WT: wild-type HEK293
PBS-T: PBS with 0.02% Tween 20
TCA: trichloroacetic acid
qPCR: quantitative PCR
DEGs: differentially expressed genes
Padj: P-adjusted
GEO: Gene Expression Omnibus
PFA: paraformaldehyde
OTM: Olive Tail Moment
RRP1B: rRNA processing protein 1 homolog B
BXDC2/BRX1: ribosome biogenesis protein BRX1 homolog
RBM28: RNA-binding protein 28
NUFIP2: nuclear fragile X mental retardation-interacting protein 2
RPS3A: 40S ribosomal protein S3a
RNA-seq: high-throughput sequencing of RNA
HeLa FUS KO: HeLa cells with depletion of FUS

## References

[1] Andersen JS, Lam YW, Leung AK, Ong SE, Lyon CE, Lamond AI (2005). Nucleolar proteome dynamics. Nature.

[2] Barral PM, Rusch A, Turnell AS, Gallimore PH, Byrd PJ, Dobner T (2005). The interaction of the hnRNP family member E1B-AP5 with p53. Febs Lett.

[3] Blackford AN, Jackson SP (2017). ATM, ATR, and DNA-PK: The Trinity at the Heart of the DNA Damage Response. Mol Cell.

[4] Blackwell DL, Fraser SD, Caluseriu O, Vivori C, Tyndall AV, Lamont RE (2022). Hnrnpul1 controls transcription, splicing, and modulates skeletal and limb development in vivo. G3-Genes Genom Genet.

[5] Caldarola S, De Stefano MC, Amaldi F, Loreni F (2009). Synthesis and function of ribosomal proteins-fading models and new perspectives. The FEBS journal.

[6] Callen E, Di Virgilio M, Kruhlak MJ, Nieto-Soler M, Wong N, Chen HT (2013). 53BP1 mediates productive and mutagenic DNA repair through distinct phosphoprotein interactions. Cell.

[7] Cerqueira AV, Lemos B (2019). Ribosomal DNA and the Nucleolus as Keystones of Nuclear Architecture, Organization, and Function. Trends in genetics: TIG.

[8] Chen DY, Huang S (2001). Nucleolar components involved in ribosome biogenesis cycle between the nucleolus and nucleoplasm in interphase cells. J Cell Biol.

[9] Ciesiolka A, Stroynowska-Czerwinska A, Joachimiak P, Ciolak A, Kozlowska E, Michalak M (2021). Artificial miRNAs targeting CAG repeat expansion in ORFs cause rapid deadenylation and translation inhibition of mutant transcripts. Cell Mol Life Sci.

[10] Correll CC, Bartek J, Dundr M (2019). The Nucleolus: A Multiphase Condensate Balancing Ribosome Synthesis and Translational Capacity in Health, Aging and Ribosomopathies. Cells.

[11] Coute Y, Burgess JA, Diaz JJ, Chichester C, Lisacek F, Greco A (2006). Deciphering the human nucleolar proteome. Mass spectrometry reviews.

[12] de Jager M, van Noort J, van Gent DC, Dekker C, Kanaar R, Wyman C (2001). Human Rad50/Mre11 is a flexible complex that can tether DNA ends. Mol Cell.

[13] Dobin A, Davis CA, Schlesinger F, Drenkow J, Zaleski C, Jha S (2013). STAR: ultrafast universal RNA-seq aligner. Bioinformatics.

[14] Emmott E, Hiscox JA (2009). Nucleolar targeting: the hub of the matter. EMBO reports.

[15] Farley KI, Surovtseva Y, Merkel J, Baserga SJ (2015). Determinants of mammalian nucleolar architecture. Chromosoma.

[16] Farley-Barnes KI, Ogawa LM, Baserga SJ (2019). Ribosomopathies: Old Concepts, New Controversies. Trends in genetics: TIG.

[17] Floutsakou I, Agrawal S, Nguyen TT, Seoighe C, Ganley AR, McStay B (2013). The shared genomic architecture of human nucleolar organizer regions. Genome research.

[18] Gabler S, Schutt H, Groitl P, Wolf H, Shenk T, Dobner T (1998). E1B 55-kilodalton-associated protein: a cellular protein with RNA-binding activity implicated in nucleocytoplasmic transport of adenovirus and cellular mRNAs. Journal of virology.

[19] Gadgil A, Walczak A, Stepien A, Mechtersheimer J, Nishimura AL, Shaw CE (2021). ALS-linked FUS mutants affect the localization of U7 snRNP and replication-dependent histone gene expression in human cells. Scientific reports.

[20] Griesbach E, Schlackow M, Marzluff WF, Proudfoot NJ (2021). Dual RNA 3'-end processing of H2A.X messenger RNA maintains DNA damage repair throughout the cell cycle. Nature communications.

[21] Grummt I (2013). The nucleolus-guardian of cellular homeostasis and genome integrity. Chromosoma.

[22] Gurunathan G, Yu Z, Coulombe Y, Masson JY, Richard S (2015). Arginine methylation of hnRNPUL1 regulates interaction with NBS1 and recruitment to sites of DNA damage. Scientific reports.

[23] Harding SM, Boiarsky JA, Greenberg RA (2015). ATM Dependent Silencing Links Nucleolar Chromatin Reorganization to DNA Damage Recognition. Cell reports.

[24] Hinsby AM, Kiemer L, Karlberg EO, Lage K, Fausboll A, Juncker AS (2006). A wiring of the human nucleolus. Mol Cell.

[25] Hoch NC, Hanzlikova H, Rulten SL, Tetreault M, Komulainen E, Ju L (2017). XRCC1 mutation is associated with PARP1 hyperactivation and cerebellar ataxia. Nature.

[26] Hong Z, Jiang J, Ma J, Dai S, Xu T, Li H (2013). The role of hnRPUL1 involved in DNA damage response is related to PARP1. PloS one.

[27] Huang S (2002). Building an efficient factory: where is pre-rRNA synthesized in the nucleolus?. J Cell Biol.

[28] Hustedt N, Durocher D (2016). The control of DNA repair by the cell cycle. Nature cell biology.

[29] Ideue T, Adachi S, Naganuma T, Tanigawa A, Natsume T, Hirose T (2012). U7 small nuclear ribonucleoprotein represses histone gene transcription in cell cycle-arrested cells. Proceedings of the National Academy of Sciences of the United States of America.

[30] Korsholm LM, Gal Z, Lin L, Quevedo O, Ahmad DA, Dulina E (2019). Double-strand breaks in ribosomal RNA genes activate a distinct signaling and chromatin response to facilitate nucleolar restructuring and repair. Nucleic acids research.

[31] Korsholm LM, Gal Z, Nieto B, Quevedo O, Boukoura S, Lund CC (2020). Recent advances in the nucleolar responses to DNA double-strand breaks. Nucleic acids research.

[32] Kruhlak M, Crouch EE, Orlov M, Montano C, Gorski SA, Nussenzweig A (2007). The ATM repair pathway inhibits RNA polymerase I transcription in response to chromosome breaks. Nature.

[33] Langhendries JL, Nicolas E, Doumont G, Goldman S, Lafontaine DL (2016). The human box C/D snoRNAs U3 and U8 are required for pre-rRNA processing and tumorigenesis. Oncotarget.

[34] Larsen DH, Hari F, Clapperton JA, Gwerder M, Gutsche K, Altmeyer M (2014). The NBS1-Treacle complex controls ribosomal RNA transcription in response to DNA damage. Nature cell biology.

[35] Levone BR, Lenzken SC, Antonaci M, Maiser A, Rapp A, Conte F (2021). FUS-dependent liquid-liquid phase separation is important for DNA repair initiation. J Cell Biol.

[36] Li ZF, Lam YW (2015). A New Rapid Method for Isolating Nucleoli. Methods Mol Biol.

[37] Liao Y, Smyth GK, Shi W (2014). featureCounts: an efficient general purpose program for assigning sequence reads to genomic features. Bioinformatics.

[38] Love MI, Huber W, Anders S (2014). Moderated estimation of fold change and dispersion for RNA-seq data with DESeq2. Genome Biol.

[39] Mangan H, Gailin MO, McStay B (2017). Integrating the genomic architecture of human nucleolar organizer regions with the biophysical properties of nucleoli. Febs Journal.

[40] Marnef A, Finoux AL, Arnould C, Guillou E, Daburon V, Rocher V (2019). A cohesin/HUSH- and LINC-dependent pathway controls ribosomal DNA double-strand break repair. Genes & development.

[41] Martin M (2011). Web 2.0-based e-learning. Brit J Educ Technol.

[42] Martinez-Macias MI, Moore DAQ, Green RL, Gomez-Herreros F, Naumann M, Hermann A (2019). FUS (fused in sarcoma) is a component of the cellular response to topoisomerase I-induced DNA breakage and transcriptional stress. Life Sci Alliance.

[43] Matos-Perdomo E, Machin F (2019). Nucleolar and Ribosomal DNA Structure under Stress: Yeast Lessons for Aging and Cancer. Cells.

[44] Mooser C, Symeonidou IE, Leimbacher PA, Ribeiro A, Shorrocks AMK, Jungmichel S (2020). Treacle controls the nucleolar response to rDNA breaks via TOPBP1 recruitment and ATR activation. Nature communications.

[45] Nemeth A, Grummt I (2018). Dynamic regulation of nucleolar architecture. Curr Opin Cell Biol.

[46] Pendle AF, Clark GP, Boon R, Lewandowska D, Lam YW, Andersen J (2005). Proteomic analysis of the Arabidopsis nucleolus suggests novel nucleolar functions. Molecular biology of the cell.

[47] Perez-Riverol Y, Bai JW, Bandla C, Garcia-Seisdedos D, Hewapathirana S, Kamatchinathan S (2022). The PRIDE database resources in 2022: a hub for mass spectrometry-based proteomics evidences. Nucleic acids research.

[48] Phair RD, Misteli T (2000). High mobility of proteins in the mammalian cell nucleus. Nature.

[49] Polo SE, Blackford AN, Chapman JR, Baskcomb L, Gravel S, Rusch A (2012). Regulation of DNA-End Resection by hnRNPU-like Proteins Promotes DNA Double-Strand Break Signaling and Repair. Mol Cell.

[50] Raczynska KD, Ruepp MD, Brzek A, Reber S, Romeo V, Rindlisbacher B (2015). FUS/TLS contributes to replication-dependent histone gene expression by interaction with U7 snRNPs and histone-specific transcription factors. Nucleic acids research.

[51] Ran FA, Hsu PD, Wright J, Agarwala V, Scott DA, Zhang F (2013). Genome engineering using the CRISPR-Cas9 system. Nat Protoc.

[52] Raska I, Shaw PJ, Cmarko D (2006). New insights into nucleolar architecture and activity. International review of cytology.

[53] Schofer C, Weipoltshammer K (2018). Nucleolus and chromatin. Histochem Cell Biol.

[54] Scully R, Panday A, Elango R, Willis NA (2019). DNA double-strand break repair-pathway choice in somatic mammalian cells. Nat Rev Mol Cell Bio.

[55] Sharma V, Khurana S, Kubben N, Abdelmohsen K, Oberdoerffer P, Gorospe M (2015). A BRCA1-interacting lncRNA regulates homologous recombination. EMBO reports.

[56] Siebenwirth C, Greubel C, Drexler GA, Reindl J, Walsh DWM, Schwarz B (2019). Local inhibition of rRNA transcription without nucleolar segregation after targeted ion irradiation of the nucleolus. J Cell Sci.

[57] Sleeth KM, Sorensen CS, Issaeva N, Dziegielewski J, Bartek J, Helleday T (2007). RPA mediates recombination repair during replication stress and is displaced from DNA by checkpoint signalling in human cells. Journal of molecular biology.

[58] Stochaj U, Weber SC (2020). Nucleolar Organization and Functions in Health and Disease. Cells.

[59] Szaflarski W, Lesniczak-Staszak M, Sowinski M, Ojha S, Aulas A, Dave D (2022). Early rRNA processing is a stress-dependent regulatory event whose inhibition maintains nucleolar integrity. Nucleic acids research.

[60] Tsekrekou M, Stratigi K, Chatzinikolaou G (2017). The Nucleolus: In Genome Maintenance and Repair. Int J Mol Sci.

[61] Tubbs A, Nussenzweig A (2017). Endogenous DNA Damage as a Source of Genomic Instability in Cancer. Cell.

[62] van Sluis M, McStay B (2015). A localized nucleolar DNA damage response facilitates recruitment of the homology-directed repair machinery independent of cell cycle stage. Genes & development.

[63] Velichko AK, Razin SV, Kantidze L (2021). DNA Damage Response in Nucleoli. Mol Biol+.

[64] Villacis LN, Wong MS, Ferguson LL, Hein N, George AJ, Hannan KM (2018). New Roles for the Nucleolus in Health and Disease. Bioessays.

[65] Vitor AC, Huertas P, Legube G, de Almeida SF (2020). Studying DNA Double-Strand Break Repair: An Ever-Growing Toolbox. Front Mol Biosci.

[66] Warmerdam DO, van den Berg J, Medema RH (2016). Breaks in the 45S rDNA Lead to Recombination-Mediated Loss of Repeats. Cell reports.

[67] Warner JR (1999). The economics of ribosome biosynthesis in yeast. Trends in biochemical sciences.

[68] Wickham H, Spathis A, Chin C, Ryan R, Booth S (2016). Practical management of chronic breathlessness. Bmj.

