# Supplementary material for: The Novel Role of hnRNP UL1 in Human Cell Nucleoli
Source: Int J Biol Sci. 2022 Jul 18;18(13):4809–23. doi: 10.7150/ijbs.75084 (PMC9379416; doi:10.7150/ijbs.75084)
Supplement: Supplementary file 1 — Supplementary figures and table. [file ijbsv18p4809s1.pdf]

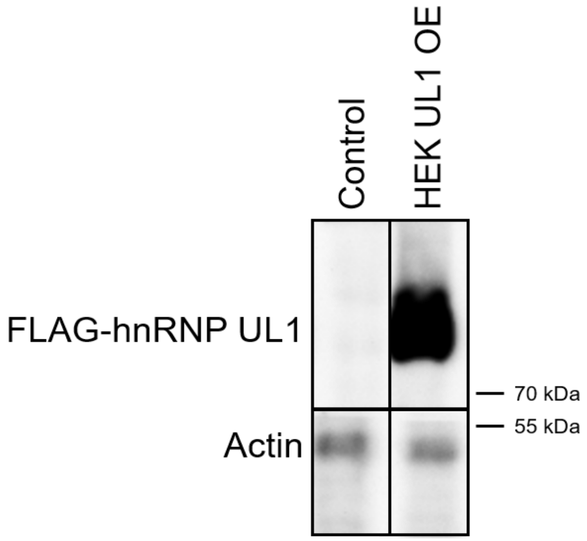

Supplementary Figure S1. Confirmation of the overexpression of FLAG-hnRNP UL1 in HEK UL1 OE cells. Western blotting followed by immunostaining using an anti-FLAG antibody. HEK WT cells were used as controls. Actin was used as a loading control.

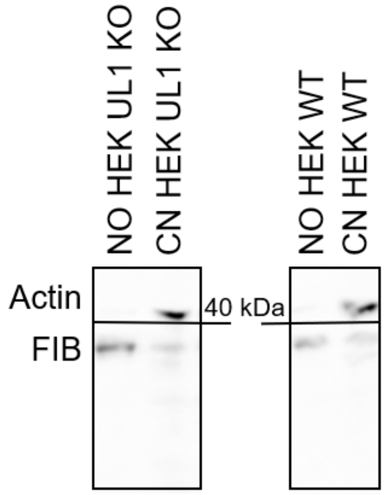

Supplementary Figure S2. Quality of isolated nucleoli from HEK UL1 KO and HEK WT cells. An anti-fibrillarin antibody (FIB) was used to detect fibrillarin, a marker of the NO fraction, while anti-actin was used to detect actin, a marker of the CN fraction.

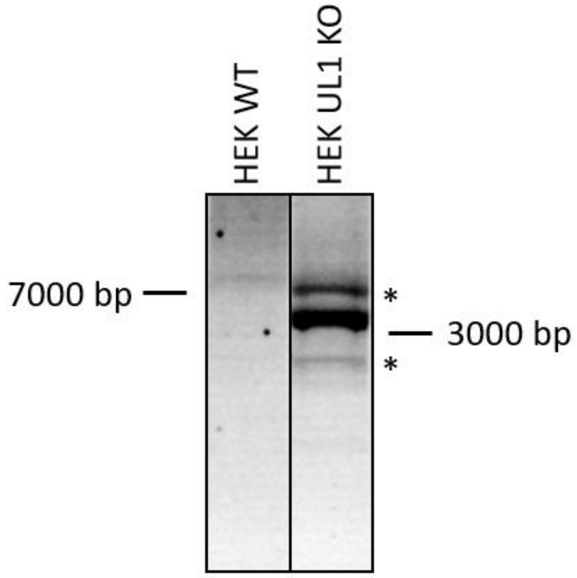

Supplementary Figure S3. Confirmation of hnRNP UL1 knockout in the HEK293T cell line. For PCR, primers flanking the fragment of the sequence deleted from the *hnRNPUL1* gene were used. After electrophoresis, the expected product with a length of 7500 nucleotides was visible only in HEK WT cells. In HEK UL1 KO cells, a product of 3600 nucleotides was visible after removing part of the *hnRNPUL1* gene sequence. *- nonspecific products.

Supplementary Table S1. hnRNP UL1 is involved in the expression of rDNA genes. rDNA genes representing 5S, 5.8S, 18S, 28S and 45S in both fractions. The fold change values indicate statistically significant (p value<0.05) changes in ribosomal gene expression in HEK UL1 KO cells relative to HEK WT cells.

GeneID	baseMean	Fold Change	p.value	p.adj	Fraction
RNA5SP174	2,639652483	0,093756	0,021534	NA	NO
RNA5SP174_1	2,639652483	0,093756	0,021534	NA	NO
RNA5SP52	2,780315849	0,17247	0,04725	NA	NO
RNA5SP358	8,327307614	0,186378	0,007575	NA	NO
RNA5SP273	10,28763423	0,286508	0,006362	0,025892	CN
RNA5-8SN1_1	95769,36236	0,297807	4,56E-11	1,34E-09	CN
RNA5-8SP8	92224,52912	0,299762	1,74E-09	3,74E-08	CN
RNA5-8SP9	93956,86347	0,304285	2,25E-09	4,75E-08	CN

RNA5-8SN1	99119,28002	0,305774	1,83E-08	3,26E-07	CN
RNA5-8SP10	96356,68146	0,305929	1,91E-07	2,81E-06	CN
RNA5SP358	8,983447886	0,334097	0,046785	0,12518	CN
RNA5-8SN3_1	89558,55004	0,372812	1,73E-21	2,02E-19	CN
RNA5-8SN2	85377,60844	0,377437	8,90E-07	1,12E-05	CN
RNA5SP74	111,7623806	0,378222	0,004721	0,02022	CN
RNA5-8SN3	90174,09109	0,378359	2,45E-06	2,76E-05	CN
RNA5-8SN5	86467,55446	0,3792	1,49E-07	2,25E-06	CN
RNA5SP336	213,6014358	0,382174	0,011705	0,042428	CN
RNA5-8SN4	88008,42441	0,411023	3,20E-16	2,00E-14	CN
RNA5SP463	36,63417027	0,418861	0,000916	0,005021	CN
RNA5SP481	176,1300573	0,42284	0,004188	0,071797	NO
RNA5SP514	33,00492196	0,423738	0,017921	0,155779	NO
RNA5SP452	15,54742946	0,428957	0,033235	NA	NO
RNA5SP452	19,8178189	0,45113	0,014121	0,049394	CN
RNA5S1_1	50,94657009	0,451417	0,0017	0,008543	CN
RNA5SP388	21,97622974	0,453121	0,023573	NA	NO
RNA5SP226	1199,702548	0,477277	0,007477	0,097746	NO
RNA5SP514	35,31856082	0,479777	0,003696	0,016492	CN
RNA5-8SP2	194,8914171	0,492964	0,033554	0,212363	NO
RNA5SP22	36,23984565	0,49943	0,029162	0,198582	NO
RNA5SP242	211,130031	0,507998	4,54E-06	4,84E-05	CN
RNA5SP382	61,34860108	0,509006	0,004297	0,018671	CN
RNA5SP71	54,2514101	0,518031	0,020664	0,167242	NO

RNA5SP149	22225,59106	0,525397	0,001559	0,007935	CN
RNA5-8SN3	52744,81405	0,525759	0,030141	0,201622	NO
RNA5-8SN5	47495,85271	0,52679	0,036464	0,219659	NO
RNA5-8SN3_1	50755,36011	0,529616	0,037189	0,221622	NO
RNA5-8SP2	241,0065559	0,537449	4,05E-05	0,000338	CN
RNA5SP336	124,5963642	0,543561	0,02536	0,184509	NO
RNA5SP506	310,3483873	0,54575	0,019982	0,164843	NO
RNA5SP199	1071,535935	0,546713	2,98E-07	4,18E-06	CN
RNA5SP145	23216,21074	0,549881	0,007889	0,030768	CN
RNA5-8SN4	49655,88647	0,551966	0,044216	0,242201	NO
RNA5SP389_1	28874,92964	0,552589	0,013394	0,047237	CN
RNA5SP389	28890,13621	0,552769	0,013426	0,047317	CN
RNA5SP353	28,30610412	0,554949	0,027986	0,08478	CN
RNA5SP429	823,2944164	0,564243	0,033337	0,096991	CN
RNA5SP506	326,475616	0,583667	0,000141	0,001002	CN
RNA5SP335	144,0520377	0,591656	0,004731	0,020247	CN
RNA5SP161	616,545565	0,594543	0,049391	0,256214	NO
RNA18SN3_1	1329605,513	0,596534	0,03277	0,210427	NO
RNA5SP161	664,2613499	0,601554	4,79E-06	5,05E-05	CN
RNA5S1	202,7226005	0,606118	0,000708	0,00403	CN
RNA5S10	202,7226005	0,606118	0,000708	0,00403	CN
RNA5S11	202,7226005	0,606118	0,000708	0,00403	CN
RNA5S12	202,7226005	0,606118	0,000708	0,00403	CN
RNA5S13	202,7226005	0,606118	0,000708	0,00403	CN

RNA5S14	202,7226005	0,606118	0,000708	0,00403	CN
RNA5S15	202,7226005	0,606118	0,000708	0,00403	CN
RNA5S16	202,7226005	0,606118	0,000708	0,00403	CN
RNA5S17	202,7226005	0,606118	0,000708	0,00403	CN
RNA5S2	202,7226005	0,606118	0,000708	0,00403	CN
RNA5S3	202,7226005	0,606118	0,000708	0,00403	CN
RNA5S4	202,7226005	0,606118	0,000708	0,00403	CN
RNA5S5	202,7226005	0,606118	0,000708	0,00403	CN
RNA5S6	202,7226005	0,606118	0,000708	0,00403	CN
RNA5S7	202,7226005	0,606118	0,000708	0,00403	CN
RNA5S8	202,7226005	0,606118	0,000708	0,00403	CN
RNA28SN1_1	971084,8395	0,612369	0,027813	0,194087	NO
RNA45SN3_1	2388683,306	0,6154	0,036117	0,218729	NO
RNA5SP86	65,79887453	0,616947	0,022733	0,07208	CN
RNA5SP19	61,47249058	0,619798	0,018622	0,061474	CN
RNA5SP226	1216,562734	0,620129	0,030018	0,089491	CN
RNA28SN1	1019822,894	0,623565	0,035479	0,217159	NO
RNA5SP263	321,2502475	0,628599	0,001918	0,009473	CN
RNA28SN5	1078174,854	0,629581	0,038225	0,22491	NO
RNA28SN3_1	1095112,589	0,64214	0,044984	0,243611	NO
RNA5SP352	194,2956415	0,644582	0,00259	0,012233	CN
RNA5SP325	129,0073428	0,645102	0,003655	0,016331	CN
RNA28SN4	1131840,758	0,645523	0,048442	0,253671	NO
RNA5SP355	381,6358449	0,688346	0,001296	0,006793	CN

RNA5SP259	203,1662227	0,699156	0,005829	0,02409	CN
RNA18SP4	1937613,645	0,702189	0,000862	0,004772	CN
RNA5SP502	147,0476978	0,703515	0,038772	0,108611	CN
RNA5SP99	309,952062	0,713696	0,00869	0,033212	CN
RNA5SP387	171,4496708	0,71973	0,044793	0,121181	CN
RNA5S9	415,8925443	0,731034	0,024936	0,077452	CN
RNA18SN3	2506339,157	0,738389	0,004723	0,02022	CN
RNA18SN2	2412270,153	0,741561	0,004933	0,020944	CN
RNA18SN1	1639718,143	0,743712	0,006016	0,024732	CN
RNA18SP3	1415510,974	0,746208	0,006001	0,02469	CN
RNA18SN3_1	2453019,488	0,750491	0,004038	0,017739	CN
RNA18SN4	2016471,222	0,760372	0,006933	0,027728	CN
RNA18SN5	1419125,21	0,78828	0,017536	0,058558	CN
RNA45SN3_1	4109132,44	0,793464	0,02107	0,067932	CN
RNA45SN3	4237521,359	0,793614	0,027184	0,082796	CN
RNA45SN4	3744350,979	0,803431	0,029961	0,089358	CN
RNA45SN2	3926619,35	0,803773	0,037811	0,106568	CN
RNA45SN1_1	2894221,604	0,803897	0,042528	0,116529	CN
RNA18SP	16848,71019	1,192551	0,016118	0,054816	CN

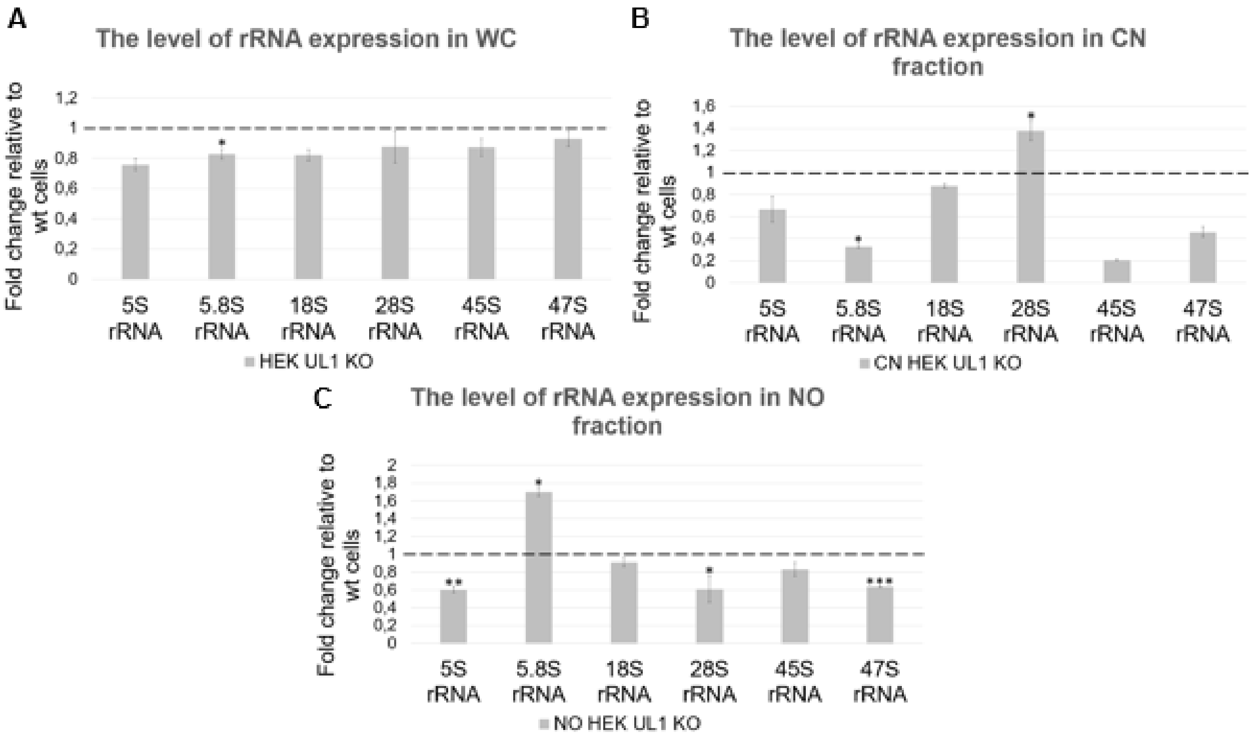

Supplementary Figure S4. Regulation of the expression of rDNA genes and ribosomal protein genes by hnRNP UL1. (A-C) rDNA gene expression was analyzed in (A) WCs and in (B) CN and (C) NO fractions isolated from HEK WT and HEK UL1 KO cells. The error bars represent the SDs of three biological replicates. The p values were calculated using Student's t test, and the statistical significance is represented as follows: *P ≤ 0.05; **P ≤ 0.01; ***P ≤ 0.001.

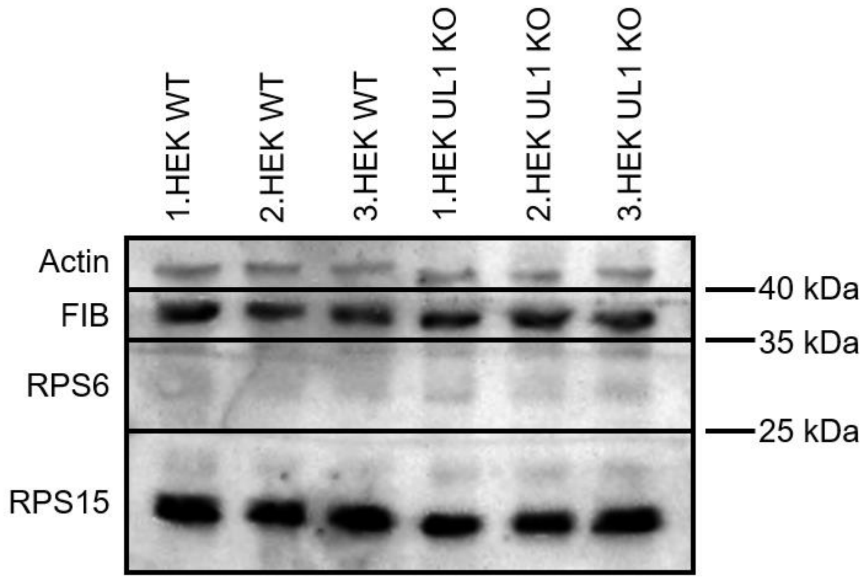

Supplementary Figure S5. Levels of ribosomal proteins (RPS6 and RPS15) and the nucleolar marker fibrillarin (FIB) in HEK UL1 KO cells compared to HEK WT cells. Western blotting and immunostaining were performed using anti-RPS6, anti-RPS15, and anti-FIB antibodies. Actin was used as a loading control. The experiment was performed in three biological replicates.

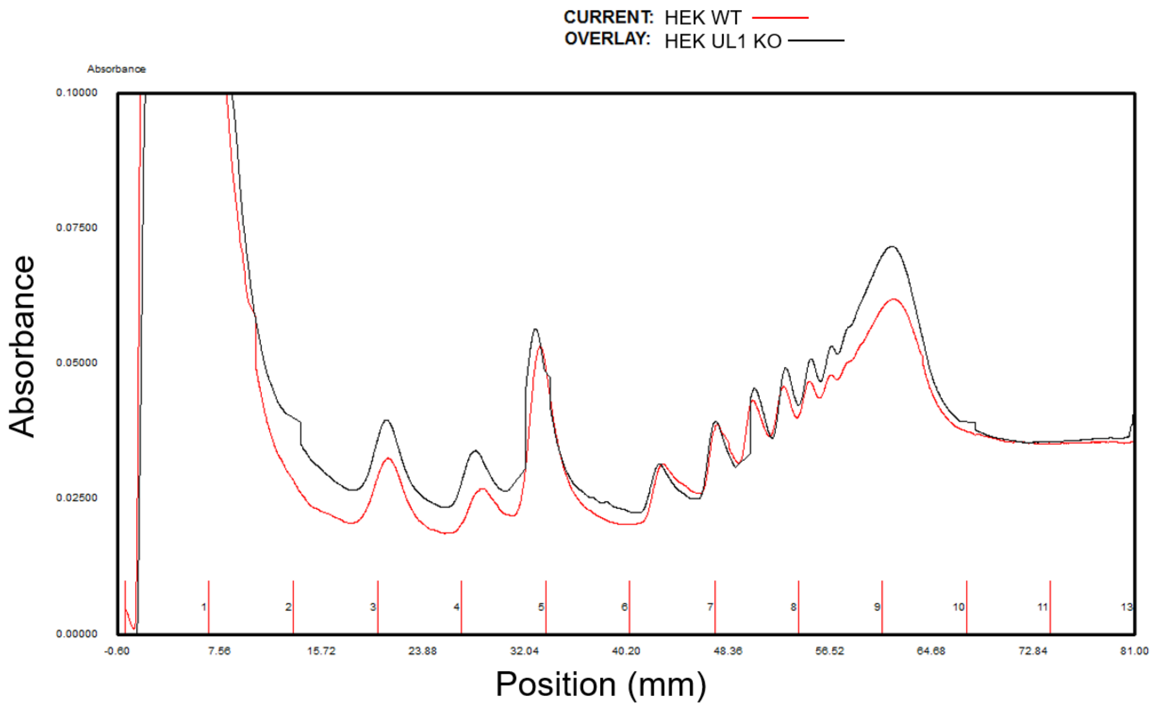

Supplementary Figure S6. Polysome profiling in HEK UL1 KO cells compared to HEK WT cells. The absorbance profiles of polysomes isolated from HEK WT (red line) and HEK UL1 KO (black line) cells were overlapped.

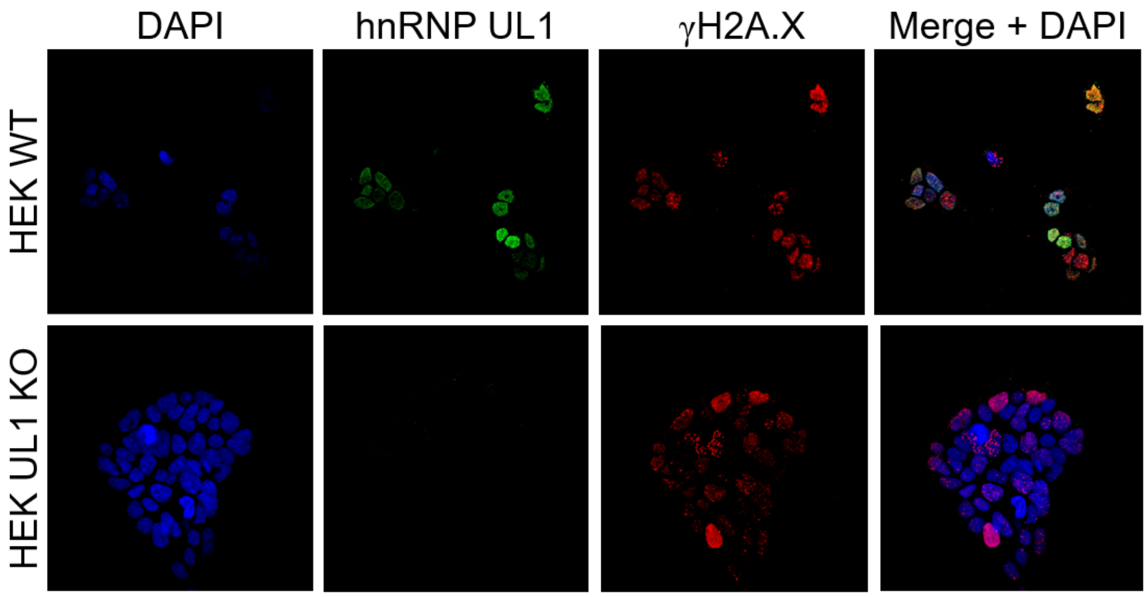

Supplementary Figure S7. Examination of γH2A.X levels in HEK UL1 KO cells relative to HEK WT cells. Cells were stained using anti-γH2A.X and anti-hnRNP UL1 antibodies. DAPI was used for nuclear staining. Scale bars: 20 µm.

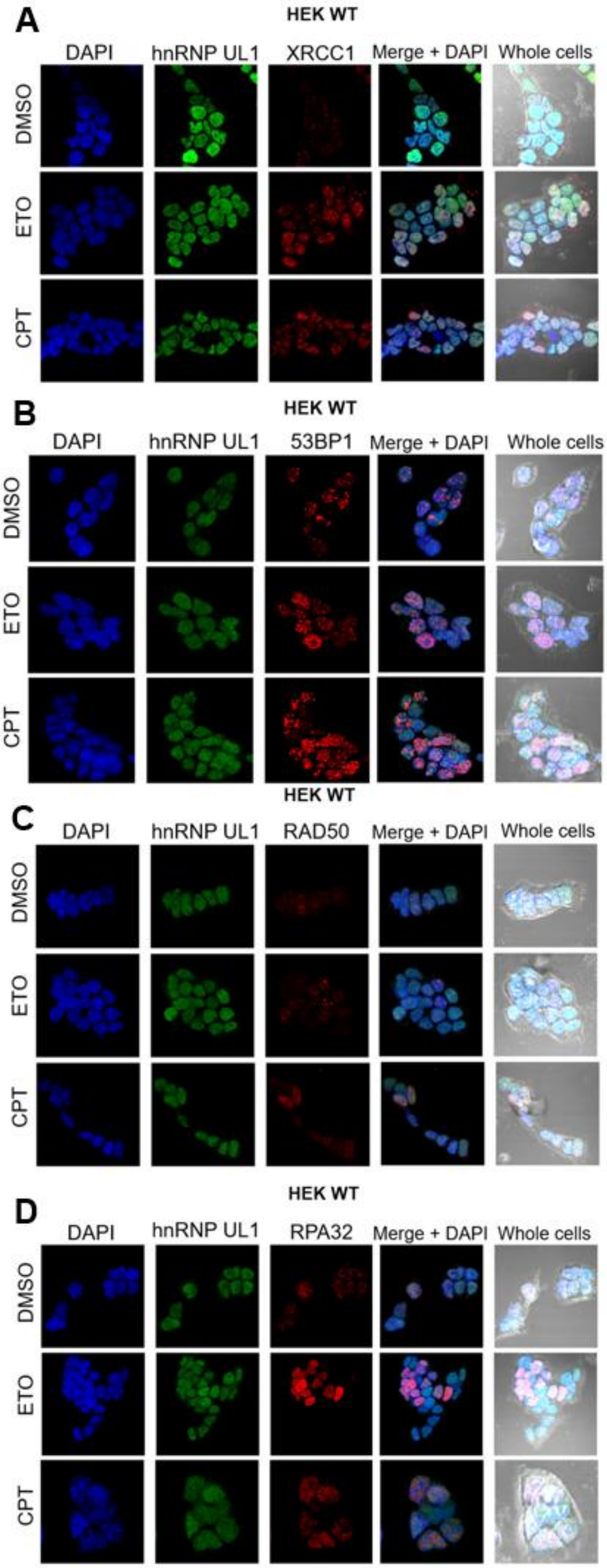
A
HEK WT
DAPI
hnRNP UL1
XRCC1
Merge + DAPI
Whole cells
DMSO
ETO
CPT
B
HEK WT
DAPI
hnRNP UL1
53BP1
Merge + DAPI
Whole cells
DMSO
ETO
CPT
HEK WT
C
DAPI
hnRNP UL1
RAD50
Merge + DAPI
Whole cells
DMSO
ETO
CPT
HEK WT
D
DAPI
hnRNP UL1
RPA32
Merge + DAPI
Whole cells
DMSO
ETO
CPT

Supplementary Figure S8. Colocalization of hnRNP UL1 with known DDR markers in HEK WT cells after induced DNA damage. DNA damage in HEK WT cells was induced by ETO and CPT. (A) Staining using anti-hnRNP UL1 and anti-XRCC1 antibodies. (B) Staining using anti-hnRNP UL1 and anti-53BP1 antibodies. (C) Staining using anti-hnRNP UL1 and anti-RAD50 antibodies. (D) Staining using anti-hnRNP UL1 and anti-RPA32 antibodies. Cells treated with DMSO were used as negative controls. DAPI was used for nuclear staining. Scale bars: 20 µm.
